# The genome of kenaf (*Hibiscus cannabinus* L.) provides insights into bast fibre and leaf shape biogenesis

**DOI:** 10.1111/pbi.13341

**Published:** 2020-01-30

**Authors:** Liwu Zhang, Yi Xu, Xingtan Zhang, Xiaokai Ma, Lilan Zhang, Zhenyang Liao, Qing Zhang, Xuebei Wan, Yan Cheng, Jisen Zhang, Dongxu Li, Liemei Zhang, Jiantang Xu, Aifen Tao, Lihui Lin, Pingping Fang, Shuai Chen, Rui Qi, Xiuming Xu, Jianmin Qi, Ray Ming

**Affiliations:** ^1^ Key Laboratory for Genetics, Breeding and Multiple Utilization of Crops Ministry of Education/Fujian Provincial Key Laboratory of Crop Breeding by Design/College of Agriculture Fujian Agriculture and Forestry University Fuzhou China; ^2^ Experiment Station of Jute and Kenaf in Southeast China of Ministry of Agriculture and Rural Affairs/Public Platform for Germplasm Resources of Bast Fiber Crops of Fujian/Fujian International Cooperation Base of Science and Technology for Genetics, Breeding and Multiple Utilization Development of Southern Economic Crops Fujian Agriculture and Forestry University Fuzhou China; ^3^ Center for Genomics and Biotechnology of Haixia Institute of Science and Technology Fujian Agriculture and Forestry University Fuzhou China; ^4^ Department of Plant Biology University of Illinois at Urbana‐Champaign Urbana IL USA

**Keywords:** Kenaf (*Hibiscus cannabinus* L*.*), genome, leaf shape, bast fibre, domestication

## Abstract

Kenaf is an annual crop that is widely cultivated as a source of bast (phloem) fibres, the phytoremediation of heavy metal‐contaminated farmlands and textile‐relevant compounds. Leaf shape played a unique role in kenaf improvement, due to the inheritance as a single locus and the association with fibre development in typical lobed‐leaf varieties. Here we report a high‐quality genome assembly and annotation for var. ‘Fuhong 952’ with 1078 Mbp genome and 66 004 protein‐coding genes integrating single‐molecule real‐time sequencing, a high‐density genetic map and high‐throughput chromosome conformation capture techniques. Gene mapping assists the identification of a homeobox transcription factor *LATE MERISTEM IDENTITY 1* (*HcLMI1*) gene controlling lobed‐leaf. Virus‐induced gene silencing (VIGS) of *HcLMI1* in a lobed‐leaf variety was critical to induce round (entire)‐like leaf formation. Candidate genes involved in cell wall formation were found in quantitative trait loci (QTL) for fibre yield and quality‐related traits. Comparative genomic and transcriptome analyses revealed key genes involved in bast fibre formation, among which there are twice as many cellulose synthase A (CesA) genes due to a recent whole‐genome duplication after divergence from *Gossypium*. Population genomic analysis showed two recent population bottlenecks in kenaf, suggesting domestication and improvement process have led to an increase in fibre biogenesis and yield. This chromosome‐scale genome provides an important framework and toolkit for sequence‐directed genetic improvement of fibre crops.

## Introduction

Kenaf (*Hibiscus cannabinus*, 2n = 36), a diploid plant in the Malvaceae family, is one of the most important species after cotton and jute for natural fibre production (Zhang *et al.*, 2015a). Polyploidy is recognized as an influence on plant genome evolution, and as a well‐established signs of whole‐genome duplication (WGD) in many sequenced genomes, such as *Gossypium* species including *G. raimondii* (DD, D‐genome) (Paterson *et al.*, 2012), *G. arboreum* (AA, A‐genome) (Li *et al.*, 2014), *G. hirsutum* (AtDt) (Zhang *et al.*, 2015b) and *G. australe* (GG, G‐genome) (Cai *et al.*, 2019). The ploidy in *Hibiscus* varies from 2 to 16, including *H. phoeniceus* (2n = 2x = 22), *H. pedunculatus* (2n = 2x = 30), *H. syriacus* (2n = 4x = 80), *H. aspera* (2n = 8x = 72) and *H. rosasinensis* (2n = 16x = 144). Recently, a draft genome of *H. syriacus* was assembled with a genome size of 1.75 Gb (Kim *et al.*, 2017). In contrast to seed fibre in cotton, bast (phloem) fibre is derived from the stem bark of plants such as kenaf, jute (*Corchorus* L.), hemp (*Cannabis sativa*), ramie (*Boehmeria nivea*) and flax (*Linum usitatissimum*). Although the genomes of the seed fibre species *G. arboretum* (Li *et al.*, 2014), *G. raimondii* (Paterson *et al.*, 2012) and *G. hirsutum* (Zhang *et al.*, 2015b) have been sequenced. However, genomic information on bast fibre species is limited and molecular biology research progresses slowly. The sequencing of the kenaf genome will enhance understanding of the genetic mechanism on bast fibre development, as it has for jute (Islam *et al.*, 2017). Kenaf was presumably domesticated in Africa and exhibits a wide range of adaptation to different climates and soils (Zhang *et al.*, 2015a). Kenaf has gained much attention worldwide due to the high biomass yields from kenaf that can be used to produce paper, rope, building materials, livestock feed, absorbents and so on. The annual global production of jute, kenaf and allied fibre generates a farm value of ∼US$2.3 billion (http://www.fao.org/faostat/en/#data/QC).

Leaves are the primary source of photoassimilate in crops. Remarkable phenotypic difference exists for leaf shape in kenaf, including two types of round (entire) and lobed leaves. Leaf shape in kenaf is an important trait that affects canopy architecture, yield and other plant attributes. A typical lobed‐leaf kenaf cultivar produces a lower canopy of round leaves before transitioning to an upper canopy of tri‐, penta‐ and septi‐lobed leaves, the growth stage that is associated with bast fibre development. Leaf shape in kenaf is unique, and breeders used a single locus to purposefully alter leaf shape among cultivars, especially hybrids. And bast fibre in kenaf makes up 35–40% of stem weight and can be processed into high‐quality industrial materials because of its low content of woody impurities and pectin (Xiong, 2008). A precise understanding of the genetic architecture underlying leaf morphology and bast fibre is critical for improving the fibre yield and quality of climate‐resilient kenaf varieties.

In the present study, we sequenced and assembled the genome of the elite *H. cannabinus* var. ‘Fuhong 952’, which is a major cultivar in China (Zhang *et al.*, 2015a) and identified key genes involved in the development of bast fibre and leaf shape. Moreover, we resequenced 20 core cultivars from 70 kenaf germplasm to reveal origin and selective sweeps under improvement. These genomic resources will be the foundation for accelerating the genetic improvement of kenaf.

## Results

### Sequencing, assembly and annotation

**Figure 1:**
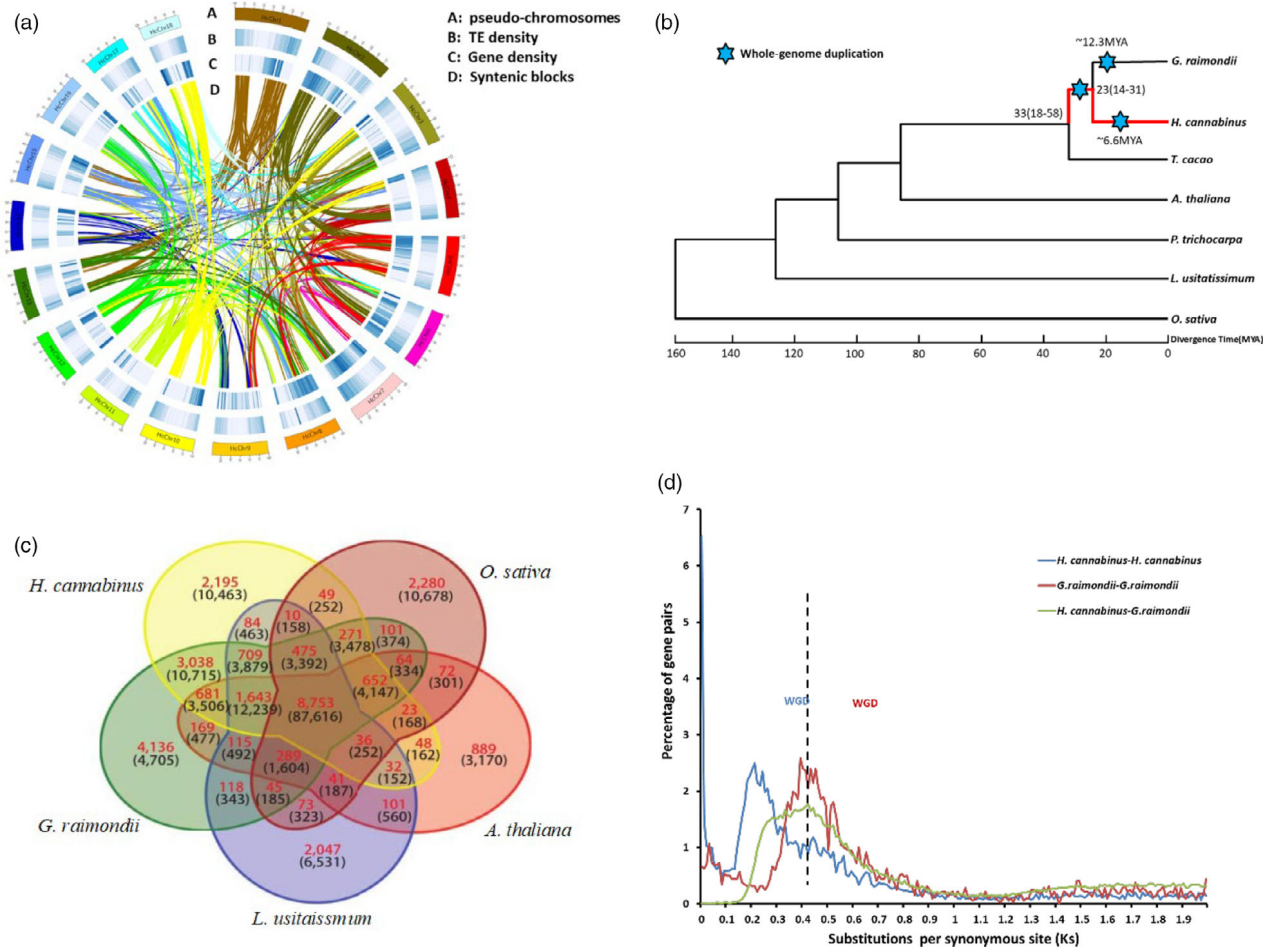
Comparative analyses and evolution of the H. cannabinus genome. (a) Basic genome information, including pseudo‐chromosomes (A), transposable elements density (B), gene density (C) and syntenic block (D), in H. cannabinus. (b) Phylogenetic analysis of seven sequenced plant genomes with O. sativa as an outgroup. H. cannabinus diverged from H. cannabinus‐G. raimondii common ancestor 23 million years ago (Mya) with the confidence interval ranging from 14 to 31 Mya. Blue stars indicate whole‐genome duplication (WGD). (c) Venn diagram of unique and shared gene families among five representative genomes. The analysis was performed with gene families common to the five genomes. (d) Ks distributions of all homologous gene pairs in the *H. cannabinus* and *G. raimondii* genomes. The *y*‐axis shows the percentage of two‐member gene clusters.

*Hibiscus cannabinus* var. ‘Fuhong 952’ was chosen for genome sequencing. The genome size of *H. cannabinus* was estimated at 1000 Mbp using flow cytometry with *Arabidopsis thaliana* genome as a reference (Figure S1). A high‐quality *H. cannabinus* genome was obtained by incorporating single‐molecule real‐time (SMRT) long reads, Illumina short reads, chromatin conformation capture technology (Hi‐C) as well as a high‐density genetic map. Appropriate 77 Gb (~80 × coverage) raw SMRT data were generated using the PacBio Sequel System. The contig‐level assembly was performed on PacBio long reads using the CANU package (Koren *et al.*, 2017) (Table S1). The resulting assembly contains 1078 Mbp sequences, similar to the estimated genome size based on flow cytometry, with contig N50 of 2.73 Mbp and the longest contig length of 18.2 Mbp (Table 1). Hi‐C libraries yielded 212 million 150‐bp paired‐end Illumina reads (Table S2). Karyotype analysis reveals 18 pairs of chromosomes in *H. cannabinus* (Figure S2). Based on the number of chromosomes, these paired‐end Hi‐C reads were uniquely mapped onto the assembly contigs and grouped into 18 pseudo‐chromosomes (Burton *et al.*, 2013) (Figure 1a, Figure S3, Table S3).

**Table 1:** Global statistics of the *Hibiscus cannabinus* genome assembly and annotation

Genome features	Contig level	Chromosomal level
Total size of assembly (Mb)	1084	1078
Number of chromosomes†		18
Number of contigs	2176	1990
Longest length (Mb)	18.2	79
N50 (Mb)	2.73	56
GC content (%)		37.6
Transposable elements (%)		67.83
Gene density‡		0.61
miRNAs		131

^†^ FISH (fluorescence *in situ* hybridization) shown in Figure S2.

^‡^ Gene density expressed in number of genes per 10 kb and based on total chromosomal length (1078 Mb for *Hibiscus cannabinus*).

To increase the accuracy of the assembly, Illumina HiSeq short reads (Table S4) were recruited to further improve the assembly using the Pilon program (Walker *et al.*, 2014). A total of 121.75 million (99.3%) reads were mapped to the assembly (Table S5). The quality of the assembly was further assessed by mapping RNA‐Seq reads to the genome. A total of 441 970 of 485 096 (91.11%) transcripts could be aligned to at least one contig with 90% sequence identity. We detected 97.38%, 99.21% or 99.80% of transcripts with minimum lengths of 500, 1000 or 2000 bp, respectively, which could be aligned in our kenaf genome assembly (Table S6). Moreover, 234 (94.4%) gene models out of the 248 ultra‐conserved core eukaryotic genes (CEGs) from CEGMA analysis (Parra *et al.*, 2007) (Table S7), and 1375 (95.5%) out of 1440 conserved genes from BUSCO analysis (Simao *et al.*, 2015) (Table S8) were completely recalled in our assembly. These results indicate a high‐quality assembly and a high level of completeness. A high‐resolution genetic map based on 3828 evenly distributed single‐nucleotide polymorphism (SNP) markers derived from a ‘Zanyin No. 1’ × ‘Fuhong 952’ F_2_ of 390 individuals showed that 99.44% (1072 out of 1078 Mbp) of the assembled genome was anchored and oriented to 18 pseudo‐chromosomes (Table S9; Figure S4).

Based on this reference genome of *H. cannabinus*, 66 004 genes were annotated by combining *ab initio* gene prediction, homologous protein searches and assembly of RNA‐Seq reads. The average gene length in *H. cannabinus* is 3226 bp, and the number of exons is 5.78 at average (Table S10). Compared with *G. raimondii* genome (Paterson *et al.*, 2012), *H. cannabinus* genome contains the average gene length at 3225.7 bp and the average exon number per gene at 5.78. In most *H. cannabinus* chromosomes, genes were enriched in the sub‐telomeric regions, while transposable elements were distributed mainly in gene‐poor regions (Figure 1a). To identify the putative functions of genes, these annotated kenaf genes were compared against the protein sequences available at public databases from various species with an E‐value threshold of 10^−5^. Of these 66 004 kenaf genes, 53 686 (81.20%) were present in at least one published genome, including *T. cacao* (Argout *et al.*, 2011), *G. hirsutum* (Li *et al.*, 2015), *G. raimondii* (Paterson *et al.*, 2012 *)*, *A. thaliana* (Initiative, 2000; Riechmann *et al.*, 2000) or *O. sativa* (Goff *et al.*, 2002). This indicates the high accuracy of *H. cannabinus* gene predictions (Table S11). Among these kenaf genes, 46 823 (70.82%) and 45 607 (68.98%) displayed high similarity to known proteins in *T. cacao* and *G. raimondii*, respectively, which also belong to the Malvaceae. However, the number of mapped genes in *H. cannabinus* (46 822) is about twice that in *T. cacao* (18 627) and *G. raimondii* (24 935) (Table S11), which suggests a possible mechanism for the dramatic increase in the number of genes in *H. cannabinus*. A total of 131 microRNAs (miRNAs) were also identified based on the search of public miRNA databases (Table 1; Table S12). Further, 39 telomere fragments (Table S13) and 3572 centromere fragments (Table S14). 67.83% transposable elements (TEs) (Table 2) were predicted in the genome of *H. cannabinus*, which were divided into two main classes: I and II, containing 58.41% retro‐element and 8.7% DNA transposon, respectively.

**Table 2:** Summary and content analysis of different types of transposable elements in the *Hibiscus cannabinus* genome

Items	Number	Length (Mb)	Per cent of assembled genome (%)
Total repeat fraction	1 422 622	732	67.83
Class I: Retroelement	757 069	630	58.41
LTR Retrotransposon	398 688	472	43.72
Ty1/Copia [RLC]	131 768	202	18.69
Ty3/Gypsy [RLG]	159 226	203	18.86
Other	107 694	66.6	6.17
Non‐LTR Retrotransposon	261 227	135	12.52
LINE [Rlx]	217 603	129	12
SINE [RSx]	43 624	5.6	0.52
Unclassified retroelement	97 154	23.5	2.18
Class II: DNA transposon	321 833	93.8	8.7
TIR			
CMC [DTC]	10 179	3.6	0.34
hAT [DTA]	45 670	11.3	1.05
Mutator [DTM]	20 520	8.3	0.77
Tcl/Mariner [DTT]	4570	1.5	0.14
PIF/Harbinger [DTH]	2341	0.5	0.05
Other	233 983	67	0.01
Helitron	727	0.1	0.01
Tandem repeats	335 985	34.8	3.23
Unknown	41 075	11.1	1.03

### Phylogenetic analysis and whole‐genome duplications

We examined the evolutionary relationship between kenaf and six other sequenced plant genomes, including representatives from the Malvids (*T. cacao* (Argout *et al.*, 2011), *G. raimondii* (Paterson *et al.*, 2012) and *A. thaliana* (Riechmann *et al.*, 2000)), Fabids (*L. usitatissimum* (Wang *et al.*, 2012) and *P. trichocarpa* (Tuskan *et al.*, 2006)) and *O. sativa* (Goff *et al.*, 2002). Phylogenetic analysis based on a concatenated alignment of 80 single‐copy gene families from seven sequenced plant genomes supported the placement of kenaf with cacao and cotton in the Malvaceae (Figure 1b). This phylogeny also reflected the position of *H. cannabinus* within the Malvaceae and speciation between *H. cannabinus* and *G. raimondii* that occurred 14–31 million years ago (Mya). Among these sequenced plant genomes, all protein‐coding genes from five genomes (kenaf, *Arabidopsis*, rice, flax and cotton) clustered into 283 581 gene families, of which 8753 were common to these five plant genomes. Among the species‐specific gene families, 2195 are unique to *H. cannabinus* (Figure 1c). These *H. cannabinus*‐specific gene families were significantly enriched with genes related to the ‘genetic information’ process’, ‘environmental information processing’, ‘diseases’ and ‘cellular processes’ (Tables S15–S18, Figure S5) according to GO term enrichment analysis. To identify *H. cannabinus*‐specific genes involved in bast fibre formation, transcription factors were analysed based on homologies to genes reported in *Arabidopsis* (Taylor‐Teeples *et al.*, 2014; Zhao and Dixon, 2011) (Table S19). Among these, we identified 67 NAC and 47 MYB transcription factors that have been implicated in an *Arabidopsis* gene regulatory network for secondary cell wall biosynthesis (Table S19, Figure S6).

Because *H. cannabinus* and *G. raimondii* are members of the same family in different genera, the extent of gene duplications in the genomes of these related species was investigated. By calculating the synonymous substitution rates (Ks) for paralogous gene pairs, a peak at 0.2 for *H. cannabinus* and at 0.4 for *G*. *raimondii* was found (Figure 1d). The peak for *H. cannabinus* reveals that a whole‐genome duplication (WGD) occurred ∼6.6 Mya in its ancestor. These results combined with the phylogenetic analysis (Figure 1b) indicate that the WGD of *H. cannabinus* happened after divergence between *Hibiscus* and *Gossypium*.

To determine the evolution of genes after the WGD, single‐copy genes in syntenic blocks and the fates of their counterparts were identified. A total of 2517 single‐copy genes were found that had no homologous counterparts in kenaf. Among these genes, 2260 genes had been deleted from the kenaf genomic sequences and 257 genes showed frameshift mutations (Table S20). We performed GO enrichment on the repetitive genes generated by WGD and found that most of the repetitive genes after WGD are genes involved in ‘cellular process’, ‘structural molecule’ and ‘cell part’ (Figure S7).

### A *LATE MERISTEM IDENTITY 1* (*LMI1*) gene responsible for leaf shape in *H. cannabinus*

**Figure 2:**
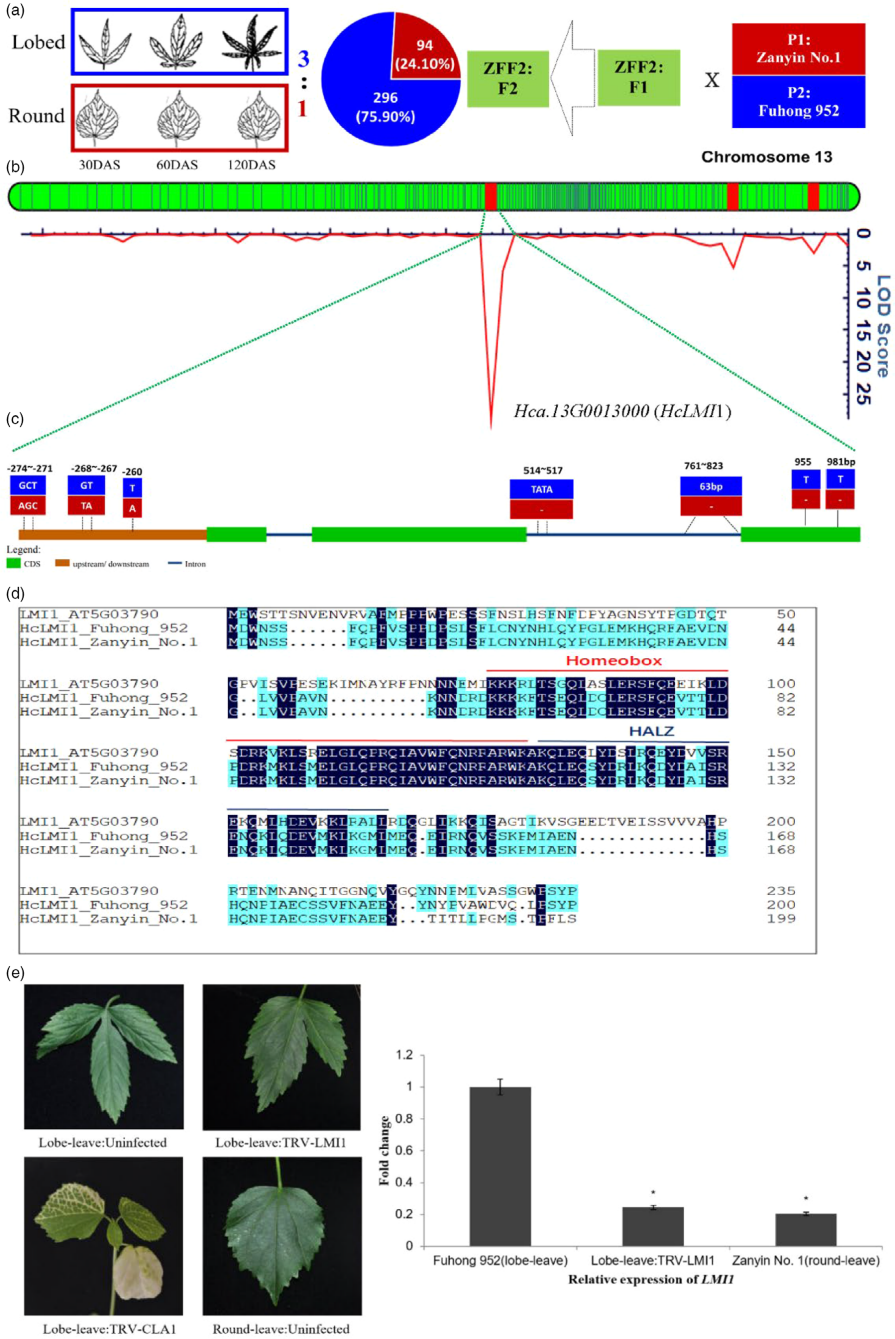
Molecular mapping of leaf shape gene (*HcLMI1*). (a) The 3:1 segregation ratio of lobed‐leaf: round‐leaf in the F_2_ population indicated a major gene controlling this trait with lobed‐leaf as dominate and round‐leaf recessive (Zhang et al. 2019). (b) LOD score for leaf shape gene mapping, based on a high‐density SNP genetic map, shown in Table S9. (c) Gene structure and variation in a candidate leaf shape gene *HcLMI1*. Exons and introns are represented by boxes and lines, respectively. The position of the causal micro‐structure variation is marked. Red and blue boxes represent P_1_‐’Zanyin No. 1’ and P_2_‐‘Fuhong 952’, respectively. (d). Amino acid comparison of *Hc LMI1* between P_1_‐’Zanyin No. 1’ and P_2_‐‘Fuhong 952’. The red line: Homeobox domain. The blue line: HALZ domain. e. Representative leaves from VIGS experiment showing the reversion to round‐like leaf shape (Lobed‐leaf: TRV‐LMI1) in the *HcLMI1* silencing treatment; Albino phenotype of *HcCLA1* silenced kenaf plants represents successfully establishment of tobacco rattle virus‐induced genes silencing (TRV‐VIGS); Relative transcript levels of candidate genes in the *HcLMI1* silenced and control Lobe‐leave plants (*n* = 3) confirmed the effective knockdown of *LMI1*. Asterisks represent statistically significant differences as determined by unpaired t tests at *P* < 0.05.

The transition of leaf shape from tri‐, to penta‐ and septi‐lobed leaves accompanies number of phloem bundles and fibrous layers during the development of typical lobed‐leaf kenaf cultivars (Figure S8 and S9). To dissect the genetic basis of leaf shape, the round‐leaved cultivar ‘Zanyin No. 1’ was crossed with two lobed‐leaf breeding cultivars, ‘Fuhong 952’ (same as the reference genome) and ‘Zhonghongma 16’. Two F_2_ populations were developed from these crosses, namely ZFF_2_ (‘Zanyin No. 1’ × ‘Fuhong 952’) with 390 individuals (Figure 2a) and ZZF_2_ (‘Zanyin No. 1’ × ‘Zhonghongma 16’) with 60 individuals (Figure S10). Chi‐square test showed that the ratio of two phenotypic genotypes of leaf shape in the two F_2_ populations fitted the Mendelian segregation ratio (3:1) of single gene, indicating a major gene controlling this trait with lobed‐leaf as dominate and round‐leaf recessive.

To map of this major gene, bulk segregant analysis (BSA), using the genome sequences with ~80× coverage with pooled DNA from 15 lobed‐leaf individuals and 15 round‐leaved individuals in the ZZF_2_ population, was conducted to localize the lobed‐leaf specific markers in contig, tig00018960, in chromosome 13 (Figure S10). The target gene was then mapped within a 10.4‐Mb interval with 42 predicted genes, using 390 ZFF_2_ individuals (Figure 2b). Further, fine mapping with reduced‐coverage genome sequences of 131 individuals in the NILs (near iso‐genic lines, BC_3_F_6_) (~5×) derived from the cross of ZFF_2_ (Figure S11) and narrowed down the locus controlling leaf shape to a region between 54.21 and 54.36 Mb within tig00018960 with six predicted genes. Of the six genes, the gene *Hca.13G0013000* (*HcLMI1*) was orthologous to the transcription factor, *LATE MERISTEM IDENTITY1* (*LMI1*, *AT5G03790*) (Vuolo *et al.*, 2018) with 51.8% protein similarity (Figure S12), encoding a homeodomain leucine zipper class I (HD‐Zip I) meristem identity regulator that acts together with *LFY* to induce *CAL* expression. Two deletions of 1‐bp, 955 and 981 bp, respectively, in the 3rd exon were found in round‐leaf *Hclmi1* compared with the gene sequence of lobed‐leaf *HcLMI1* (Figure 2c). These deletions might have resulted in a frameshift in the predicted round‐leaf *HcLMI1* that may interfere with the function of the HcLMI1 (Figure 2d). Additionally, one InDel marker (63 bp, from 761 to 823 bp in the dominant genotype) was designed within the micro‐structure variations of this candidate gene (Figure 2c), which showed complete association with leaf shape in the panel of 70 kenaf germplasm.

Moreover, quantitative (q) RT‐PCR revealed that the expression of *HcLMI1* was substantially higher in the lobed‐leaf parental variety ‘Fuhong 952’ compared with the round‐leaved parental line ‘Zanyin No. 1’ (Figure 2e). This suggests that *HcLM1* is a critical transcription factor to regulate the leaf morphogenesis in the parental cultivar ‘Fuhong 952’ with the hypothesis that silencing of *HcLM1* would reduce transcript levels and can indirectly affect a round‐like leaf shape. To confirm this hypothesis, a 251‐bp fragment of the coding sequence near 3’ UTR of *HcLMI1* was used in virus‐induced gene silencing (VIGS) (Figure 2e; Table S21). A *TRV:CLA1* treatment was used as a visible marker to verify viral infection because VIGS is sensitive to environments. Albino phenotype of *HcCLA1* silenced kenaf plants represents successfully establishment of tobacco rattle virus‐induced genes silencing (TRV‐VIGS). Silencing of *HcLMI1* in lobed‐leaf shape in the *TRV: LMI1* treatment led to a pronounced reduction in lobed‐leaf compared with uninfected (Lobed‐leaf: Uninfected) and negative controls (Lobed‐leaf: Uninfected). This proved that knocking down the *HcLMI1* transcript through VIGS was sufficient to induce round‐like leaf formation in a lobed‐leaf shape variety.

### Quantitative trait loci controlling fibre yield and quality

**Figure 3:**
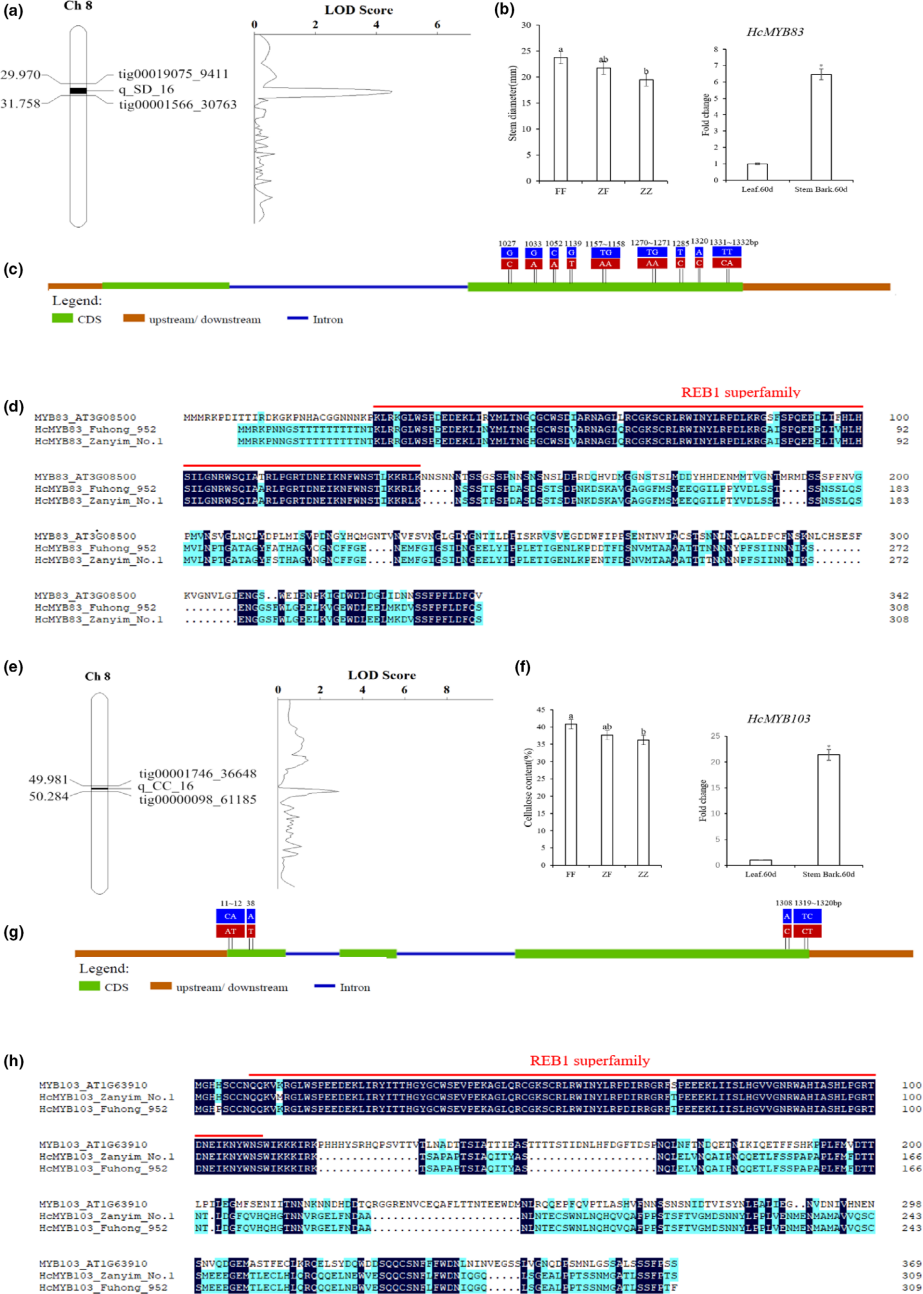
Candidate genes underlying related to bast fibre development. (a) One putative QTL of stem diameter was mapped on Chromosome 8 using the high‐density genetic linkage map. (b) Stem diameters among the three genotypes of *HcMYB83*(*Hca.08G0001750*) and expression profile of this gene between leaf.60d and stem bark.60d. (c) Gene structure and variation in the candidate gene of *HcMYB83*. Exons and introns are represented by boxes and lines, respectively. The positions of the causal micro‐structure variation are marked. Red and blue boxes represent P1‐’Zanyin No. 1’ and P2‐‘Fuhong 952’ respectively. (d) Amino acid comparison of HcMYB83 between P1‐’Zanyin No. 1’ and P2‐‘Fuhong 952’. (e) One putative QTL of cellulose content of bast fibre was mapped on Chromosome 8 using the high‐density genetic linkage map. (f) Stem diameters among the three genotypes of *HcMYB103*(*Hca.08G0028100*) and expression profile of this gene between leaf.60d and stem bark.60d. (g) Gene structure and variation in a candidate gene of *HcMYB103*. Exons and introns are represented by boxes and lines, respectively. The positions of the causal micro‐structure variation are marked. Red and blue boxes represent P1‐’Zanyin No. 1’ and P2‐‘Fuhong 952’, respectively. (h) Amino acid comparison of HcMYB103 between P1‐’Zanyin No. 1’ and P2‐‘Fuhong 952’.

To reveal candidate genes for agronomically important traits, quantitative trait loci (QTL) of 6 fibre yield and quality traits, including plant height, stem diameter, fresh bark thickness, fresh stem weight, dry bark weight and cellulose content of bast fibre, were mapped in the ZFF_2_ and their F_2:3_ populations, respectively (Table S22). A total of 112 QTLs were detected. Through BLAST searching against the reference genome using flanking DNA markers, candidate genes involved in cell wall formation, such as cellulose synthase‐like and UDP‐D‐glucuronate 4‐epimerase as well as MYBs, were found in these loci, containing 79 candidate protein‐coding genes. Notably, two MYB transcription factors, *HcMYB83* (*Hca.08G0001750*) and *HcMYB103* (*Hca.08G0028100*), that have been implicated in an *Arabidopsis* gene regulatory network for secondary cell wall biogenesis, were detected in the QTLs of stem diameter (Figure 3a) and cellulose content of bast fibre (Figure 3e), respectively. Primary functional analysis of the two MYB transcription factors promises further information on the regulation of bast fibre development.

#### HcMYB83 (Hca.08G0001750) for stem diameter

Quantitative trait loci (QTL) analysis showed that this locus explained 12.19% of phenotype variance with the LOD score of 4.52 (Figure 3a). Significant differences among the three genotypes, ZZ (‘Zanyin No. 1’), ZF (heterozygous genotype) andFF (‘Fuhong 952’), demonstrated that genetic influence on stem diameter was pervasive in the 390 ZFF_2_ individuals. Up‐regulation of this MYB gene in the stem bark.60d may cause to increase the thickness of stem diameter (Figure 3b). Sequence analysis of *HcMYB83* between the two parental lines showed that 9 base substitutions occurred at the second exon (Figure 3c). These substitutions result in a frameshift in the predicted protein of *HcMYB83*; however, the frameshift introduces threonine and aspartic acid that may alter the function of the HcMYB83 (Figure 3d).

#### *HcMYB103* (*Hca.08G0028100*) for cellulose content of bast fibre

The putative QTL, which explained 10.52% of phenotypic variance with the LOD score of 2.90, were mapped to chromosome 8 (Figure 3e). The 390 ZFF_2_ individuals could be divided into three genotypes. ZZ alleles of *HcMYB103* exhibited low cellulose content of bast fibre in contrast to FF alleles (Figure 3f). Up‐regulation of this MYB gene in stem bark.60d may result in increasing of cellulose content of bast fibre. Sequence analysis of *HcMYB103* identified four prominent polymorphisms, among which two base substitutions were located at the beginning of the coding sequence and two base substitutions at the end of the coding sequence (Figure 3g). These substitutions result in a frameshift in the predicted protein of *HcMYB103*; however, the frameshift introduces phenylalanine that may alter the function of the HcMYB103 (Figure 3h).

### Transcriptome analysis and fibre biogenesis

Transcriptome analysis of nine different tissues was conducted to identify genes that could be involved in bast fibre formation (Table S23). The penta‐lobed‐leaf stage is a vigorous vegetative stage for fibre development in *H. cannabinus* as described by Xiong (2008), namely stem bark.60d (60 days after germination) and leaf.60d, respectively (Table S24). We compared RNA‐seq data from stem bark.60d and leaf.60d at the penta‐lobed‐leaf stage (Figure S13). In transcriptional regulation of secondary cell wall (SCW) formation, *HcMYB46*, *HcMYB85*, *HcMYB58*, *HcMYB83*, *HcMYB103*, *HcSND1*, *HcSND2*, *HcSND3* and *HcNST1* (two homologous) exhibited significantly higher expression in the stem bark.60d than in leaf.60d (Table S25). In particular, some of these genes also showed distinct expression patterns between jute and kenaf, although both undergoing secondary cell wall synthesis in bast fibres. As a major regulator that is capable of activating the biosynthesis of SCW components, *AtMYB83* and *AtMYB46* are co‐expressed and functionally redundant with each other in *Arabidopsis* (McCarthy *et al.*, 2009). A significant increase in the expression of *HcMYB46* and *HcMYB83* was observed in the stem bark.60d relative to leaf.60d, indicating that *MYB46* and *MYB83* could be of primary importance in the SCW regulatory network controlling bast fibre formation in kenaf. However, the *MYB46* homologue showed little or no expression in jute fibre (Islam *et al.*, 2017).

#### Phloem fibre cell elongation

**Figure 4:**
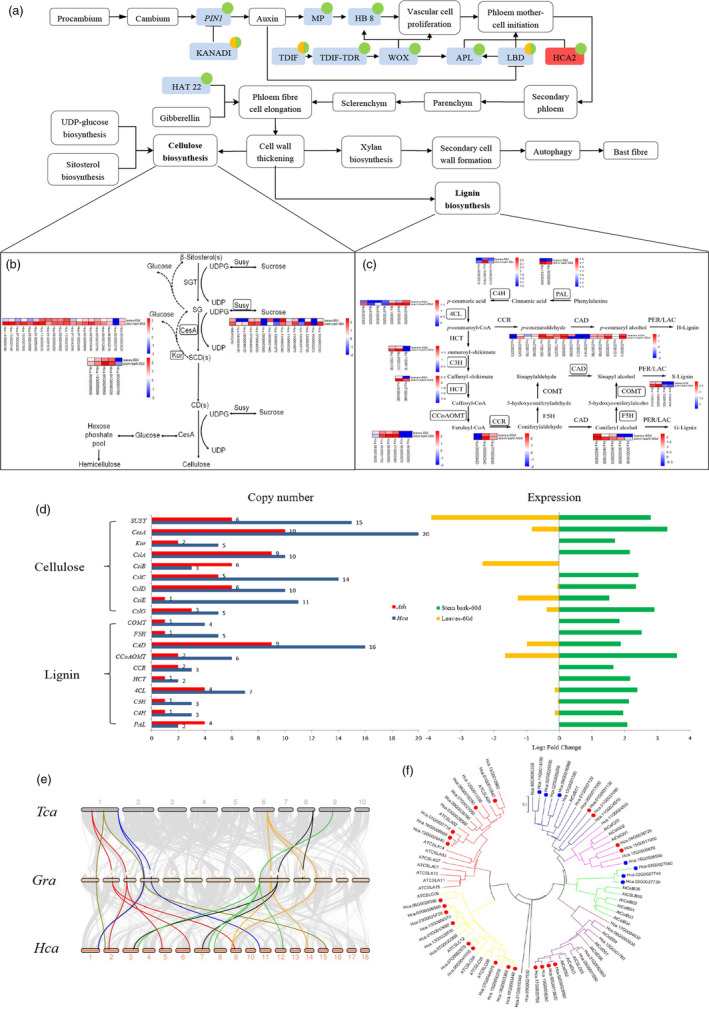
Fibre development and transcriptome comparison of genes involved in lignocellulosic biosynthesis enzymes in *H. cannabinus*. (a) Schematic representation of the fibre formation process. Fibre formation‐related genes are listed in Table S25. Blue: the more copy number in *H.ca than A.th*; Red: the same copy number in *H.ca and A.th*; Green: the gene is expressed in stem bark.60d (60 days after germination undergoing bast fibre formation); Yellow: the gene is expressed in leaf.60d. (b) The expression profile of leaf.60d and stem bark.60d in cellulose synthesis pathway at the vegetable stage of kenaf. (c) The expression profile of leaf.60d and stem bark.60d in the lignin synthesis pathway at the vegetable stage of kenaf. (d) Comparison of copy numbers of genes involved in lignocellulosic biosynthesis enzymes between *Hca* and *Ath* (is shown on the left). Comparison of relative RNA‐Seq expression of lignocellulosic biosynthetic enzymes in leaf.60d and stem bark.60d (is shown on the right). *Hca, H. cannabinus; Ath, A. thaliana.* (e) Synteny analysis among *T. cacao*, *G. raimondii* and *H. cannabinus* genomes. Grey lines in the background highlight conserved synteny blocks with more than 10 genes. Cellulose synthase A (*CesA*) genes in the synteny block are highlighted by different colours, shown in Table S26. (f) The phylogeny of cellulose synthase‐like (*Csl*) genes in *H. cannabinus* and *A. thaliana*. Different colour circles show differential expression between leaf.60d and stem bark.60d at the vegetable stage, respectively. Red circles represent up‐regulation at stem bark.60d, while blue ones indicate up‐regulation at leaf.60d.

Combined with the analysis of orthologous genes involved in plant fibre formation in *Arabidopsis* (Taylor‐Teeples *et al.*, 2014; Zhao and Dixon, 2011; Hirano *et al.*, 2013; Taylor, 2008), a model for bast fibre development in SCW formation in kenaf was proposed (Figure 4a). From this model, except for *HCA2*, copy numbers of other genes are higher than that in *Arabidopsis*, which may be caused by WGD events. Among them, *LBD1* and *KANADI* have undergone functional differentiation between stem bark.60d and leaf.60d (Figure S14, Table S25).

#### Cellulose biosynthesis

The relatively lower cellulose content and higher lignin content of bast fibres from kenaf makes them coarser. Cellulose is a major component of kenaf fibres (∼61%) and is synthesized by the cellulose synthase A (CesA) complex. Twenty CesA genes were identified in the cellulose synthesis pathway (Figure 4b, d), which showed expansions of the CesA gene families compared with those in *T. cacao* (7 *CesA* genes) and *G. raimondii* (15 CesA genes) (Islam *et al.*, 2017) (Table S26). Synteny analysis of *H. cannabinus*, *T. cacao* and *G. hirsutum* indicated that the WGD of *H. cannabinus* occurred post‐speciation, indicating that the genomic architecture of CesA genes has been shaped by the recent WGD that occurred ∼6.6 Mya (Figure 4e, Figure S15). In‐depth analysis of the expression profiles of the CesA genes between stem bark.60d and leaf.60d revealed significant qualitative transcript differences for eight homologues, highlighting possible targets for engineering high‐cellulose bast fibre in kenaf (Table S25). Expression profiles of CesA genes in different tissues and growth stages showed that SCW synthesis‐specific genes *HcCesA4* (two homologous), *HcCesA7* (three homologous) and *HcCesA8* were distinctly up‐regulated in stem bark.60d compared with other tissues, indicating their association with cellulose deposition in the SCW. However, significantly higher expression of *HcCesA1*, *HcCesA3* and *HcCesA6* in stem bark than in leaves suggests the involvement of these genes in cellulose deposition in primary cell walls (Table S25, Figure S16). Further analysis of the expression profiles of cellulose synthase‐like (*Csl*) *A/B/C/D/E/G* genes between stem bark.60d and leaf.60d (Figures 4f; Table S25) revealed that the copy number of *CslB* was less than that of *Arabidopsis.* Among them, *Csl D/E/G* have undergone functional differentiation between stem bark.60d and leaf.60d, while *CslB* was expressed only in leaf.60d rather than stem bark.60d.

#### Lignin biosynthesis

Genes encoding most of the key enzymes for lignin biosynthesis were also identified (Figure 4c; Table S25). In the lignin synthesis pathway, we compared the copy number of lignin synthesis genes in *Arabidopsis* (Figure 4d) and found that copy number of most genes except *PAL* are higher than that in *Arabidopsis.* Among them, *CAD, CCoAOMT, 4CL, C3H* and *C4H* have undergone functional differentiation between stem bark.60d and leaf.60d. The expression profiles of the lignin biosynthesis genes revealed that only a few homologous appeared to be preferentially expressed at high levels in the stem bark.60d (Figure 4c; Table S25), highlighting possible targets for engineering low‐lignin bast fibre in kenaf.

### ***Origin and selection signals under improvement of H*** *.* ***cannabinus***

Structure variations (SVs) were evaluated by whole‐genome resequencing the 20 core cultivars, which were selected from 70 kenaf germplasm (Figure S17, Table S27). An average of 11 616 SVs were detected within the coding region between each of 20 agronomically improved cultivars comparing with the reference genome (Figure S18–S21). Among these SVs, deletions (accounting for 14.97% ± 7.32% of total SVs) were found to be the most abundant, while inversions (1.97% ± 0.35%) and duplications (0.44% ± 0.09%) were the least abundant (Figure S17).

**Figure 5:**
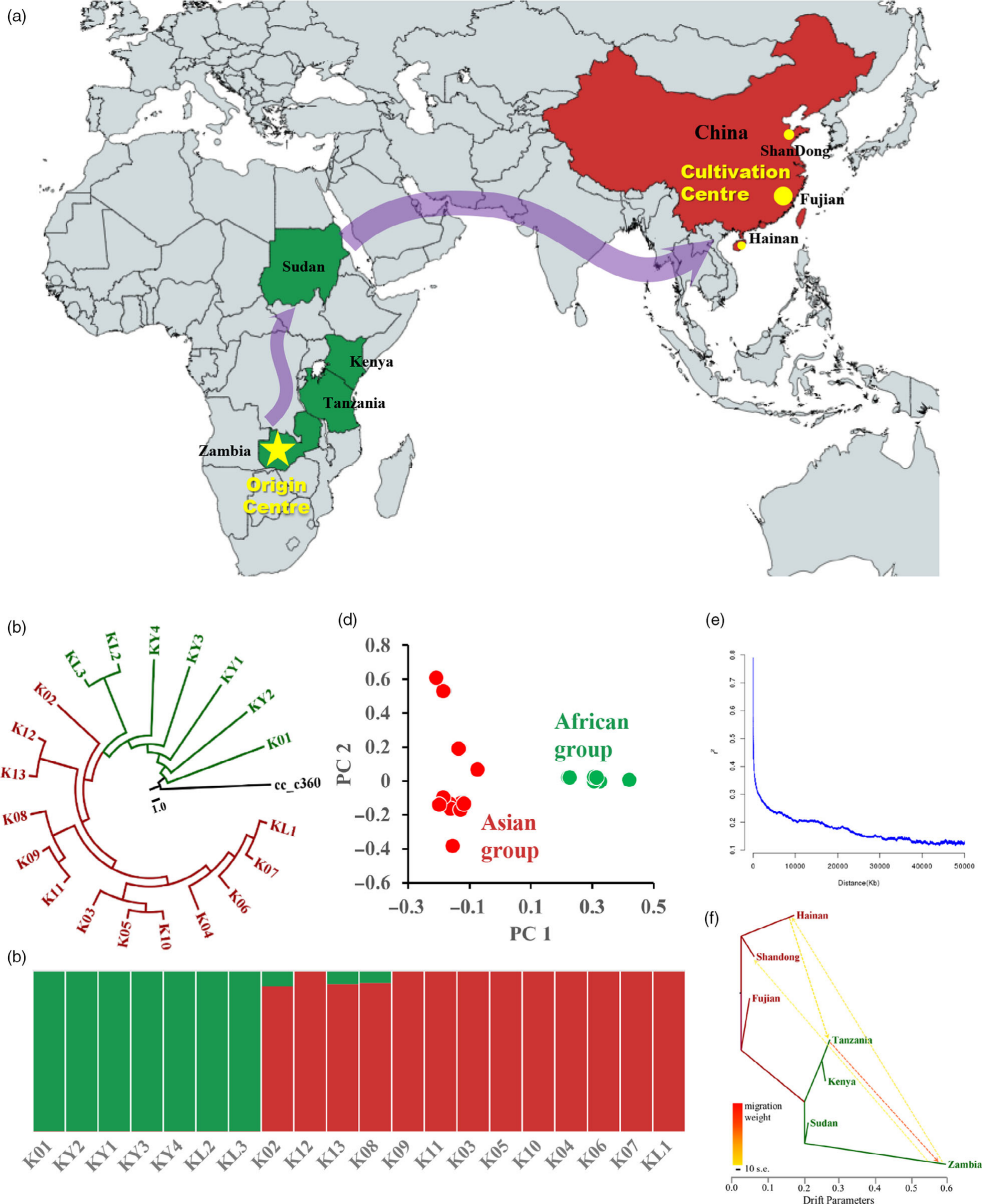
Population genomic analyses for 20 core kenaf cultivars. (a) The origin centre Zambia and evolutionary route of main cultivars from Africa to cultivation central Asia in the world. (b) Phylogenetic relationships among kenaf cultivars. (c) Population structure clustering 20 accessions into two subgroups, with optimal clusters as *K* = 2. (d) PCA shows clear separation between Africa and Asia populations. (e) LD decay with pairwise genomic distance. (f) TreeMix analysis of kenaf cultivars divided into seven geographical quadrants. The arrow indicates the direction of gene flow.

**Figure 6:**
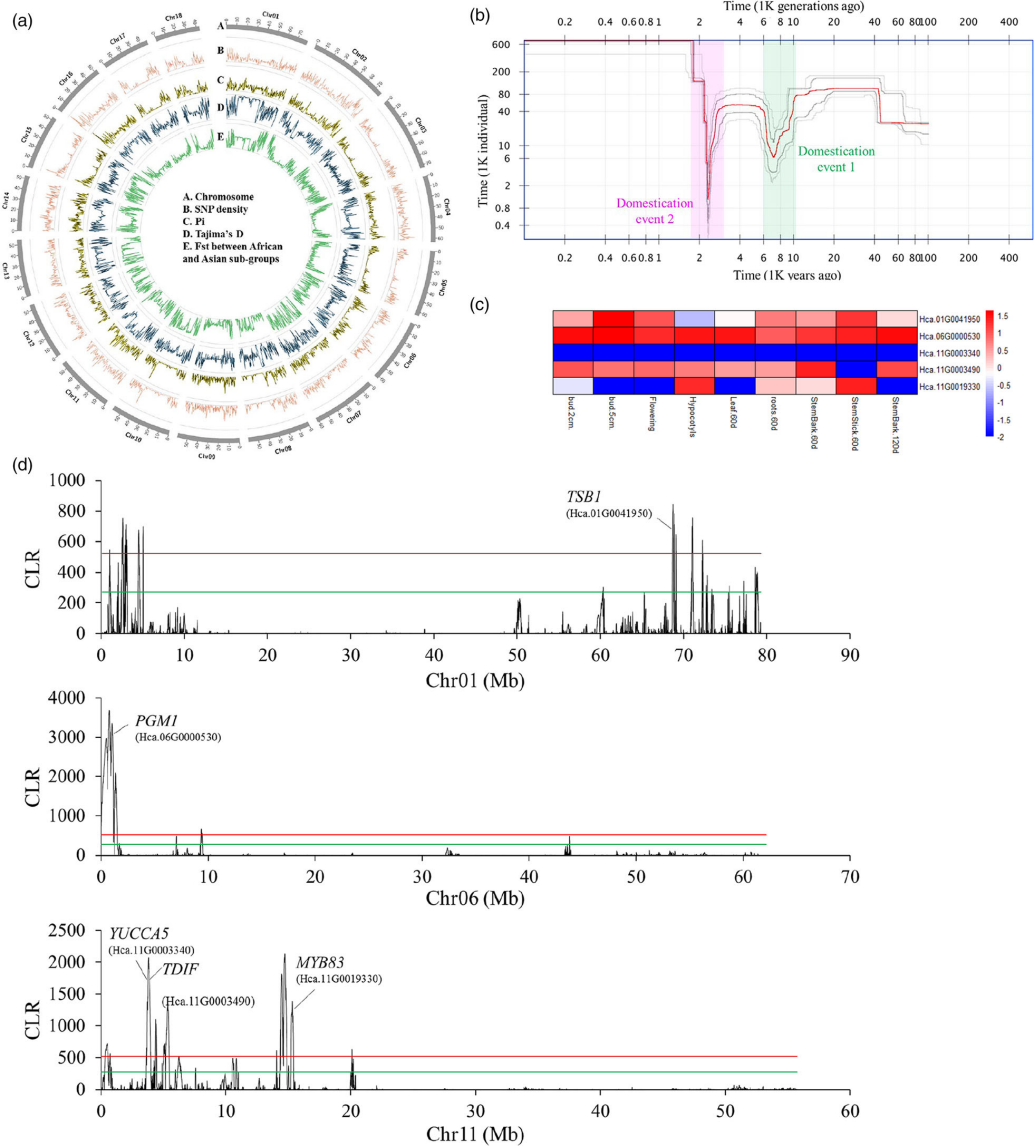
Genomic‐wide genetic diversity and selection signals under fibre improvement of *H. cannabinus*. a. Genome wide genetic diversity among core kenaf cultivars. From the outer to the centre present (A) chromosome position; (B) SNP density; (C) nucleotide diversity Pi; (D) Tajima’s D; (E) *F*_st_ between African and Asian subgroups. b. Historical effective population size (Ne) for domesticated kenaf population beginning from 101 Kya years ago to present. The plot showing kenaf population has undergone domesticated bottlenecks during two periods, including one ancient domestication event at ~10 k (green shade) years ago and a recent domestication event at ~2.3 k (pink shade) years ago. The estimate is median (red line) from 200 bootstrap replicates with 87.5% and 97.5% confidence intervals (four grey lines). (c) The fibre synthesis and metabolism‐related genes under selective sweeps regions were marked in the graphs. The fibre related genes *MYB83, TDIF, YUCCA5, PGM1* and *TSB1* may be under artificial selection during human domestication of kenaf fibres. The red solid line indicates the candidate regions above the 1% (the green solid line indicates 5%) cut‐off outlier with significant deviations from neutrality. d. Expression profile of five fibre related genes (*MYB83, TDIF, YUCCA5, PGM1* and *TSB1)* under artificial selection during human domestication of bast fibres.

For the genomic‐wide genetic diversity among 20 core kenaf cultivars, we identified 2 697 218 high confidence variants that include 2 246 488 single‐nucleotide polymorphisms (SNPs), 227 425 insertions and 223 305 deletions, averaging 2.47 variants per kb (Figure 6a). We estimated average nucleotide diversity (pi) to be 0.00072438 ± 0.000643997 and Tajima’s D value to be 1.21562 ± 1.37712 (Figure 6a). A highly positive Tajima’s D would imply population bottleneck or balancing selection in kenaf cultivars (one‐sample *t*‐test *P* < 0.05, as shown in the results of demography history). These kenaf accessions could be clustered into two main groups by principal‐component analysis (PCA) and population structure analysis (*K* = 2), among which seven are from Africa and the remaining 13 accessions from Asia (Figure 5c, d). Further, by examining the phylogenetic relationship among kenaf cultivars, we found African cultivars diverged earlier than Chinese ones. The clade in Zambia firstly diverged during kenaf population expansion (Figure 5b). We proposed the origin centre for kenaf might be Zambi or surrounding areas, and cultivars spread to Asia along the route of Southern Africa, Western Africa as shown in Figure 5a.

The *F*_st_ (average 0.365812, Figure 6a) between African and Asia subgroups shows the high genetic differentiation between them, indicating long period of historical isolation between two sub‐populations. Heat maps for introgression detection among different geographical regions showed that kenaf cultivars in Sudan, Shandong and Fujian shared ancestral alleles from Africa (Figure S22). We used TreeMix to examine the topology of relationships and migration history among populations. The direction of gene flow is from Zambia to Hainan and Shandong, then from Hainan to Tanzania, and finally from Tanzania to Zambia (Figure 5f), indicating frequently germplasm exchange between Asia and Africa during kenaf domestication. Linkage disequilibrium (LD) shows slowly decay with the *R* ^2^ approaching half of the maximum values at ~204.3 kb, which suggests a high intensity of artificial selection (Figure 5e).

The domestication history of crops usually reflected their changes in historical effective population sizes (*N_e_*) during human utilization (Tenaillon *et al.*, 2004). Stairway plot analysis with 20 core cultivars using the two‐epoch fold model (with 200 bootstraps) indicated that kenaf population beginning from ~101 K years ago to present has undergone recent bottlenecks during two periods (Figure 6b). The first estimated recent bottleneck event occurred at ~10.2 K years ago and persisted ~2K years ago. The second bottleneck started at ~3K years ago and continued ~1K years ago. Archaeobotanical evidence shows two rounds of historical domestication events at AD 6K and AD 2K years ago (Xiong, 2008). Our demography analysis showed that the periods of two minimum *Ne* occurred were close to those two periods. The first domestication event may have happened in Africa and second in both Africa and Asia countries. In addition, there is a protracted period of *Ne* decline of the first bottleneck. This may have caused by many factors, such as low temperature persistence. One possible explanation is the prolonged history of low‐intensity collection or cultivation before fully domestication at ~2K years ago.

To recover the evidence of recent selectively swept genes that might play important roles in kenaf population domestication, we analysed the same population, a set of 20 kenaf accessions, by scanning the genomic‐wide selective swept patterns using the CLR (composite likelihood ratio) statistics (Figure S23). To prevent false positives, we scanned kenaf genome in 20 kb windows of each chromosome, and strictly chose the top 1% CLR outlier regions as potential selective sweeps. A total of 926 genes undergone domesticated selection (Figure S23). These genes were significantly enriched in GO related to the ‘cell wall organization or biogenesis’, ‘structural constituent of cell walls’ and ‘cell wall’ according to GO term enrichment analysis (Figure S24, both FDR and *P* < 0.05, Fisher’s exact test). Five genes related to fibre synthesis and metabolism on three chromosomes might be selected under domestication (Figure 6c). *TBS1* and *MYB83* in kenaf showed significantly up‐regulated expression in stem bark.60d than that in leaf.60d (Figure 6d). *MYB83* is also involved in the SCW regulatory networks in jute (Islam *et al.*, 2017), suggesting parallel selection of this gene in jute and kenaf during domestication. The difference is that the homologue of *AtMYB46*, which is co‐expressed and functionally redundant with *AtMYB83* in *Arabidopsis* (McCarthy *et al.*, 2009) and kenaf (this study), showed little or no expression in jute fibres.

## Discussion

With the development of sequencing technology, more and more species completed the whole‐genome sequencing, such as *P. betuleafolia* (Dong *et al.*, 2019), *P. granatum* (Yuan *et al.*, 2018) and *M. rubra* (Jia *et al.*, 2019). We generated the first high‐quality chromosomal level genome assembly of *H. cannabinus* var. ‘Fuhong 952’ in this study. The kenaf‐specific WGD at ~6.6 Mya occurred after its divergence from *Gossypium* A or D diploid ancestral species, resulting in a dramatic increase of gene numbers compared to *G. raimondii* genome. Although *H. cannabinus* and *Gossypium* species are members of the Malvaceae family in different genera, they underwent different WGD events. In addition, LTR insertions and expansions have contributed to the doubled genome size of *H. cannabinus*.

Gene family expansion and contraction of candidate genes involved in bast fibre formation in *H. cannabinus* were detected. The WGD event in kenaf resulted in higher copy number of genes involved in fibre synthesis than in *Arabidopsis*, and some gene families showed functional differentiation. The expansion of the CesA gene families suggested that ancient paralogs can remain in the same regulatory networks for millions of years.

Leaf shape varies among plants. Understanding the genetic architecture of variation in leaf morphology in kenaf is critical to bast fibre yield improvement and plant physiology modification, because the transition of leaf shape from tri‐, to penta‐ and septi‐lobed leaves accompanies the different stages of fibre development in typical lobed‐leaf cultivars (Xiong, 2008). We identified a single locus controlling leaf shape in kenaf is governed by a transcription factor *HcLMI1* encoding an HD‐Zip transcription factor. *LMI1*‐like genes have been proposed for controlling leaf shape in other plants, such as the major leaf shape gene, *GhLMI1‐D1b*, in cotton (Andres *et al.*, 2017). Our results reinforced the notion that *LMI1*‐like genes are evolutionary hot spots for modifying leaf shape in the order of Malvales and Brassicales (Vlad *et al.*, 2014).

Kenaf was first recorded as a wild species in Africa before 6K AD. By 4K AD ago, the Republic of the Sudan domesticated kenaf for fibre crops; while China began to cultivate kenaf as a bast fibre crop at the beginning of the 20th century (Xiong, 2008). Identification of candidate genes or genomic regions for bast fibre yield and quality‐related traits provides insights into high‐yield and quality fibre formation and expedites breeding. Five genes related to fibre development were identified during domestication. Among them, *MYB83* is also involving in SCW regulatory networks of jute (Islam *et al.*, 2017), suggesting that this gene in cultivated kenaf and jute likely undergone parallel domesticated selection for fibre development.

## Experimental procedures

Materials and Methods as well as any associated references are available in the online version of the paper. *H. cannabinus* genome sequences have been deposited and gene annotation information including accession codes is in the Genome Warehouse (GWH) of BIGD (BIG Data Center) (https://bigd.big.ac.cn/gwh) with submission ID of GWHACDB00000000 (BioProject: PRJCA000871,Biosample:SAMC036340), which is also available at the Genomes of Bast Fibre Crops (GBFC), at http://gbfc.fafu.edu.cn/. All raw sequence data of RNA‐seq are accessible through the NCBI Sequence Read Archive (SRA) under accession PRJNA556928. [Correction added on 8 May 2020, after first online publication. The information on the “Experimental procedures” section has been updated.]

## Conflict of interest

The authors declare no competing financial interests.

## Author contribution

L.Z., J.Q. and R.M. jointly supervised the work. X.Z. and Z.L. performed sequencing, assembly and genome annotation. X.Z., S.C., R.Q., X.X. and X.M. performed genome analysis and physical map integration. L.Z., X.W., Y.X. and D.L. prepared DNA and RNA samples and performed PCR analysis. J.Q., J.X., A.T., P.F. and L.L. provided the homozygous seeds. L.Z., Y.C. and Q.Z. performed transcriptome and gene functional analyses. X.M. and Y.X. performed the population genomics, selection analysis and GO enrichments. L.Z. conceived the project and wrote the manuscript. L.Z. and R.M. revised the manuscript.

## Supporting information


**Figure S1** The estimated genome size of *Hibiscus cannabinus* was 1000 Mb by flow cytometry.
**Figure S2** Karyotype analysis using FISH (fluorescence in situ hybridization) in *H. cannabinus.*
**Figure S3** Hi‐ C maps of 18 chromosomes using 150 k resolution in *H. cannabinus.*
**Figure S4** High‐resolution SNP genetic map based on a F_2_ population derived from a cross between ‘Fuhong 952’ and ‘Zanyin No. 1’ in *H. cannabinus.*
**Figure S5** Frequency of KEGG pathway for specific *H. cannabinus* genes.
**Figure S6** Gene numbers of *H. cannabinus* transcription factors.
**Figure S7** GO enrichments of duplicated genes after WGD in *H. cannabinus.*
**Figure S8** Relationship between leaf shape and fibre accumulation at the different stages of growth.
**Figure S9** Different chemical components of bast fibre in *H. cannabinus.*
**Figure S10** BSA mapping of the candidate leaf shape gene (*HcLMI1*) in the population of ZZF_2_.
**Figure S11** Development of near iso‐genic lines using backcrosses.
**Figure S12** Evolutionary relationships of *LMI1*. *Ch: Cardamine hirsuta; Cr:Capsella rubella; Cg; Capsella grandiflora; Gh:Gossypium hirsutum.*
**Figure S13** Expression analysis of genes from different tissues at the various stages in *H. cannabinus.*
**Figure S14** Comparison of copy numbers of genes involved in secondary cell wall enzymes between *Hca* and *Ath*.
**Figure S15** Synthenic analysis between *H. cannabinus* and the close related species *T. cacao.*
**Figure S16** Phylogenetic analysis of cellulose synthase A (*CesA*) genes in *Arabidopsis thaliana* and *H. cannabinus.*
**Figure S17** Genetic diversity and structure variations among 20 core kenaf cultivars based on SNPs.
**Figure S18** Number of genes with deletions of 20 core *H. cannabinus* cultivars.
**Figure S19** Number of genes with insertions of 20 core *H. cannabinus* cultivars.
**Figure S20** Number of genes with inversions of 20 core *H. cannabinus* cultivars.
**Figure S21** Number of genes with tandem duplications of 20 core *H. cannabinus* cultivars.
**Figure S22** Heat maps for introgression detection among different geographical regions.
**Figure S23** Selective sweeps scanning in 20 core *H. cannabinus* cultivars.
**Figure S24** GO enrichments of selective swept genes in 20 core *H. cannabinus* cultivars.

**Table S1** PacBio sequencing for *Hibiscus cannabinus.*
**Table S2** Statistics of Hi‐C sequencing and mapping for *Hibiscus cannabinus.*
**Table S3** Statistics of chromosome‐anchored contigs for *Hibiscus cannabinus.*
**Table S4** Illumina whole‐genome shotgun sequencing for *Hibiscus cannabinus.*
**Table S5** Assessment of genome consistency for *Hibiscus cannabinus* using Illumina Hiseq.
**Table S6** Assembly assessment using Trinity de novo assembled transcripts for *Hibiscus cannabinus.*
**Table S7** Completeness of the genome based on CEGMA for *Hibiscus cannabinus.*
**Table S8** BUSSCO analysis of gene annotation for *Hibiscus cannabinus.*
**Table S9** High‐resolution genetic map based on a F_2_ population derived from a cross between ‘Fuhong 952’ and ‘Zanyin No. 1’ in *H. cannabinus.*
**Table S10** Genes annotation in *Hibiscus canabinus.*
**Table S11** The number and frequencies of genes annotated in the public databases among five sequenced species.
**Table S12** Prediction of miRNAs in *Hibiscus canabinus.*
**Table S13** Annotation of telomeres in *Hibiscus canabinus.*
**Table S14** Annotation of centromeres in *Hibiscus cannabinus.*
**Table S15** The pfam families for specific *H. cannabinus* genes.
**Table S16** KEGG pathway enrichment analysis for specific *H. cannabinus* genes.
**Table S17** Frequencies of KEGG pathway for specific *H. cannabinus* genes.
**Table S18** Transcription factors for specific*H. cannabinus* genes.
**Table S19** Frequencies of transcription factors for specific *H. cannabinus* genes.
**Table S20** Gene loss after whole‐genome duplication in *H. cannabinus.*
**Table S21** Primer sequences of genes involved in leaf shape and actin genes used for RT‐qPCR.
**Table S22** Candidate genes involved in cell wall formation for QTLs mapping.
**Table S23** RNA samples for *H. cannabinus* in this study.
**Table S24** Differentially expressed genes between stem bark and leaves for *H. cannabinus.*
**Table S25** Genes involved in bast fibre formation for *H. cannabinus.*
**Table S26** Cellulose synthase A genes (*CesA*) among the genomes of*T. cacao*, *G. raimondii* and *H. cannabinus*.
**Table S27** Structure variations of 20 core *H. cannabinus* cultivars.

**Appendix S1** Materials and Methods.

Supplementary Legends
