# Supplementary material for: The genome of kenaf (Hibiscus cannabinus L.) provides insights into bast fibre and leaf shape biogenesis
Source: Plant Biotechnol J. 2020 Jan 30;18(8):1796–809. doi: 10.1111/pbi.13341 (PMC7336286; doi:10.1111/pbi.13341)
Supplement: Supplementary file 1 — Figure S1 The estimated genome size of Hibiscus cannabinus was 1000 Mb by flow cytometry. Figure S2 Karyotype analysis using FISH (fluorescence in situ hybridization) in H. cannabinus. Figure S3 Hi‐ C maps of 18 chromosomes using 150 k resolution in H. cannabinus. Figure S4 High‐resolution SNP genetic map based on a F2 population derived from a cross between ‘Fuhong 952’ and ‘Zanyin No. 1’ in H. cannabinus. Figure S5 Frequency of KEGG pathway for specific H. cannabinus genes. Figure S6 Gene numbers of H. cannabinus transcription factors. Figure S7 GO enrichments of duplicated genes after WGD in H. cannabinus. Figure S8 Relationship between leaf shape and fibre accumulation at the different stages of growth. Figure S9 Different chemical components of bast fibre in H. cannabinus. Figure S10 BSA mapping of the candidate leaf shape gene (HcLMI1) in the population of ZZF2. Figure S11 Development of near iso‐genic lines using backcrosses. Figure S12 Evolutionary relationships of LMI1. Ch: Cardamine hirsuta; Cr:Capsella rubella; Cg; Capsella grandiflora; Gh:Gossypium hirsutum. Figure S13 Expression analysis of genes from different tissues at the various stages in H. cannabinus. Figure S14 Comparison of copy numbers of genes involved in secondary cell wall enzymes between Hca and Ath. Figure S15 Synthenic analysis between H. cannabinus and the close related species T. cacao. Figure S16 Phylogenetic analysis of cellulose synthase A (CesA) genes in Arabidopsis thaliana and H. cannabinus. Figure S17 Genetic diversity and structure variations among 20 core kenaf cultivars based on SNPs. Figure S18 Number of genes with deletions of 20 core H. cannabinus cultivars. Figure S19 Number of genes with insertions of 20 core H. cannabinus cultivars. Figure S20 Number of genes with inversions of 20 core H. cannabinus cultivars. Figure S21 Number of genes with tandem duplications of 20 core H. cannabinus cultivars. Figure S22 Heat maps for introgression detection among different geographi [file PBI-18-1796-s003.pdf]

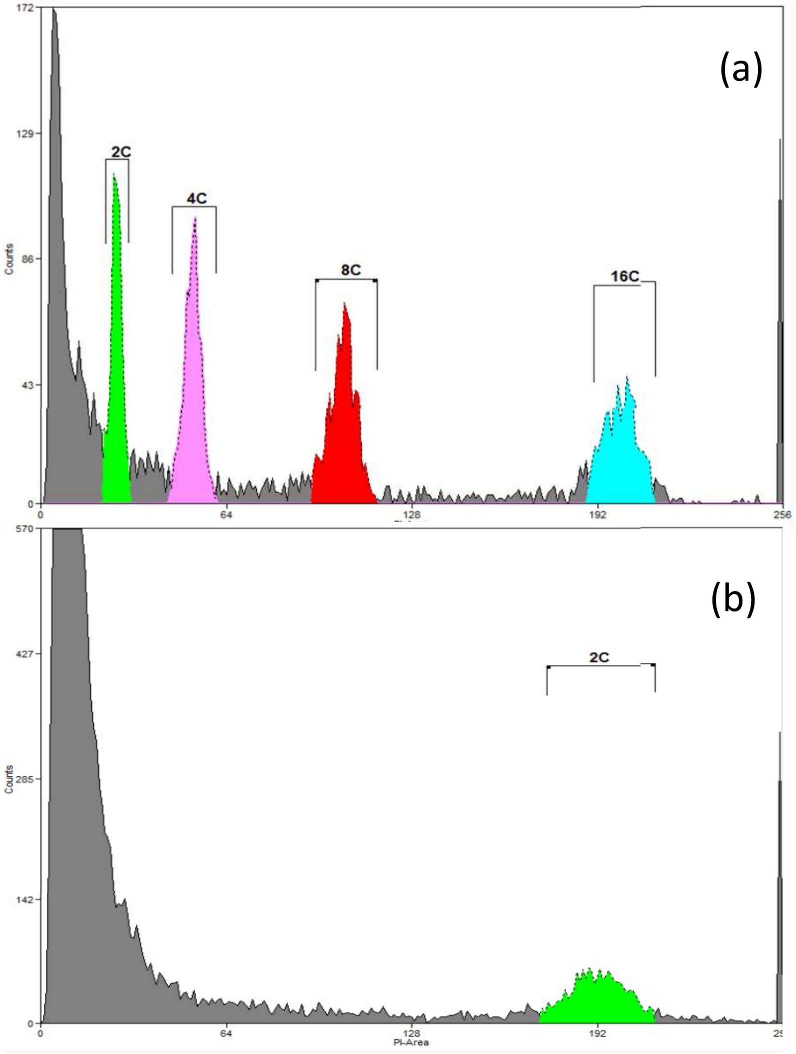


**Supplementary Figure 1** The estimated genome size of *Hibiscus cannabinus* was 1000 Mb by flow cytometry . (a)The flow cytometry figure of *A. thaliana* leaf shown four peaks: 2C=25, 4C=50, 8C=102, 16C=200. (b) The flow cytometry figure shown the 2C peaks of *Hibiscus cannabinus* leaf : 2C=195. The genome size of *A. thaliana* is 125 MB.

(a) *Arabdopsis thaliana* 2C=25

(b)*Hibiscus cannabinus* 2C=195

**Supplementary Figure 2** karyotype analysis using FISH (fluorescence in situ hybridization) in *H. cannabinus*

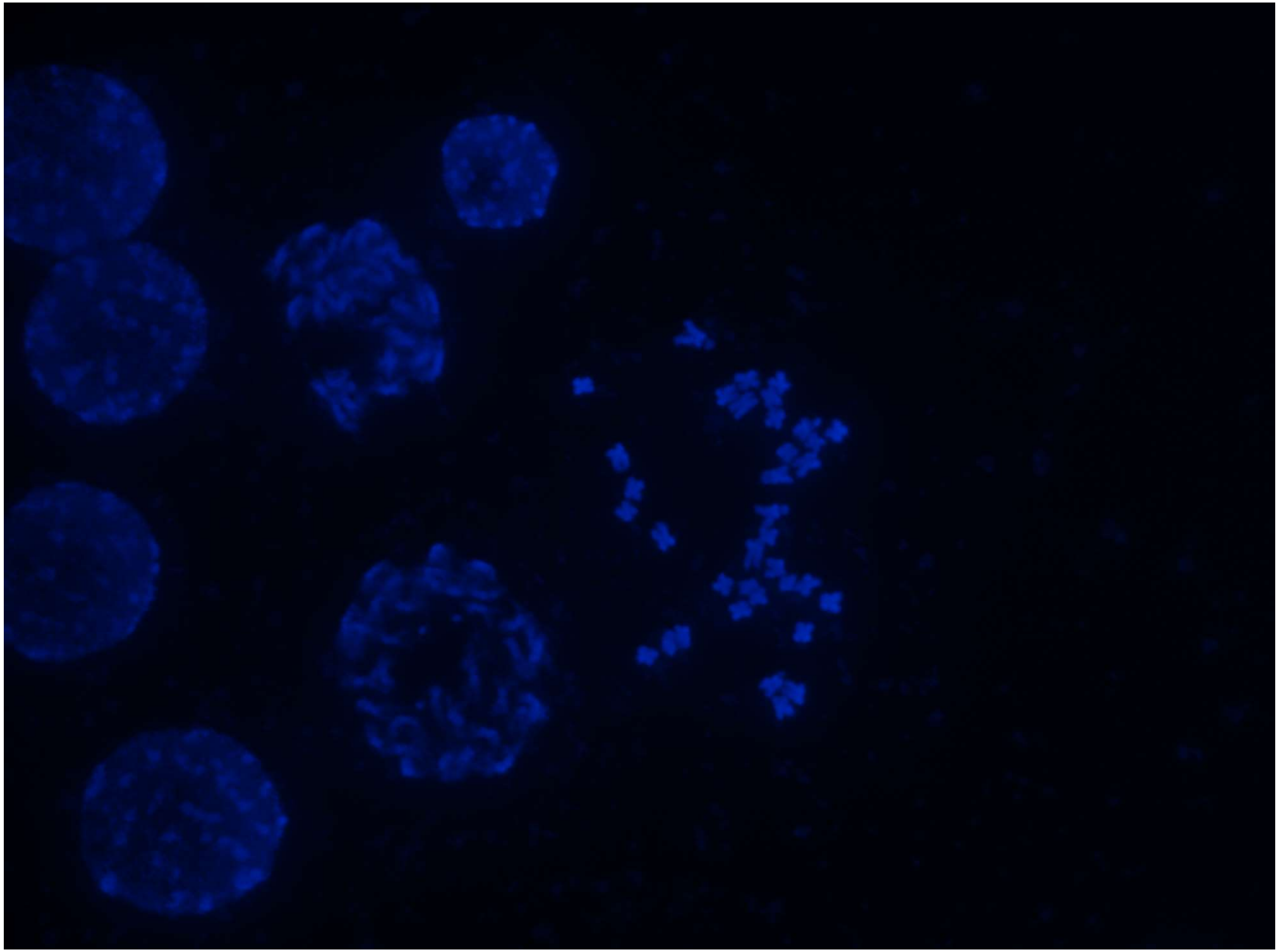

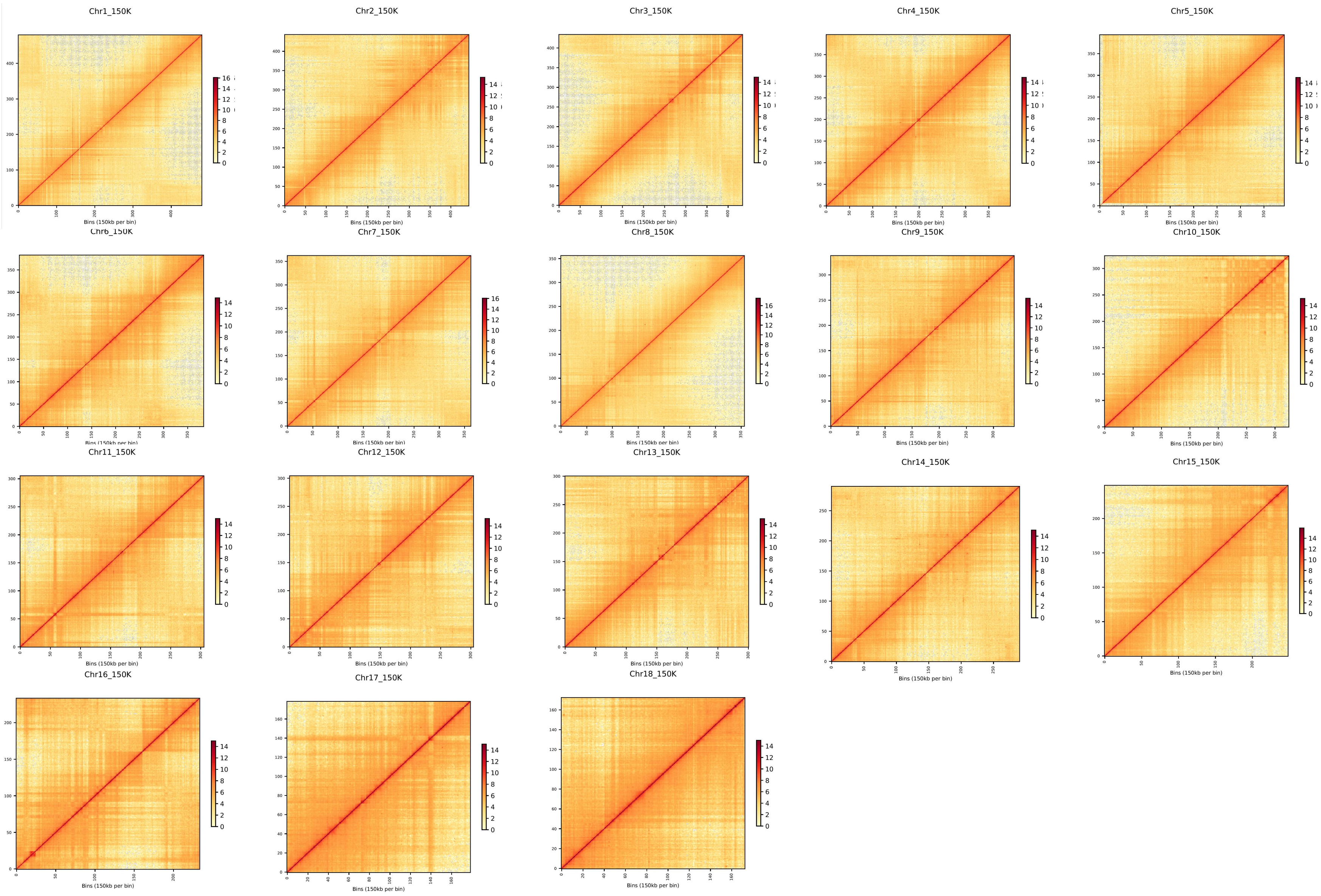


**Supplementary Figure 3** HiC maps of 18 chromosomes using 150 k resolution in *H. cannabinus*

**Supplementary Figure 4** High-resolution SNP genetic map based on a F2 population derived from a cross between ‘Fuhong 952’ and ‘Zanyin No. 1’ in *H. cannabinus*

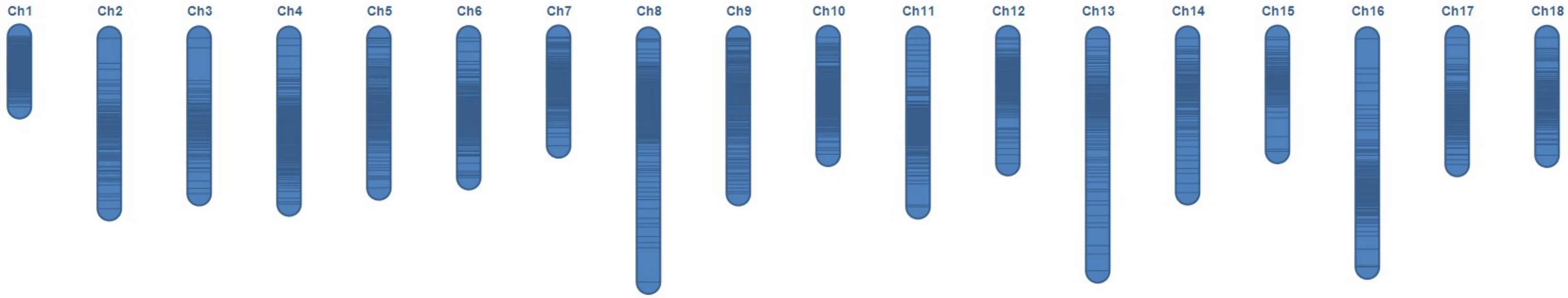

**Supplementary Figure 5** Frequency of KEGG pathway for specific *H. cannabinus* genes

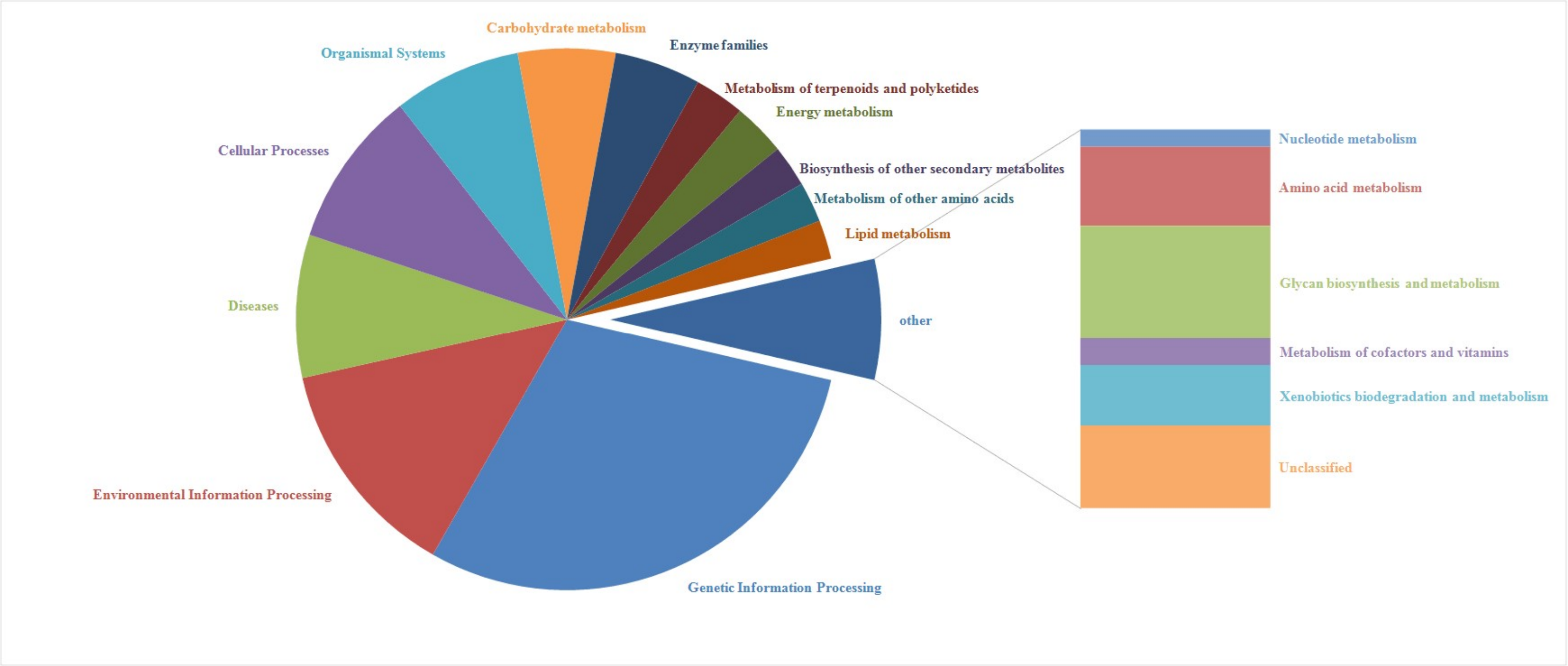

**Supplementary Figure 6** Gene numbers of *H. cannabinus* transcription factors

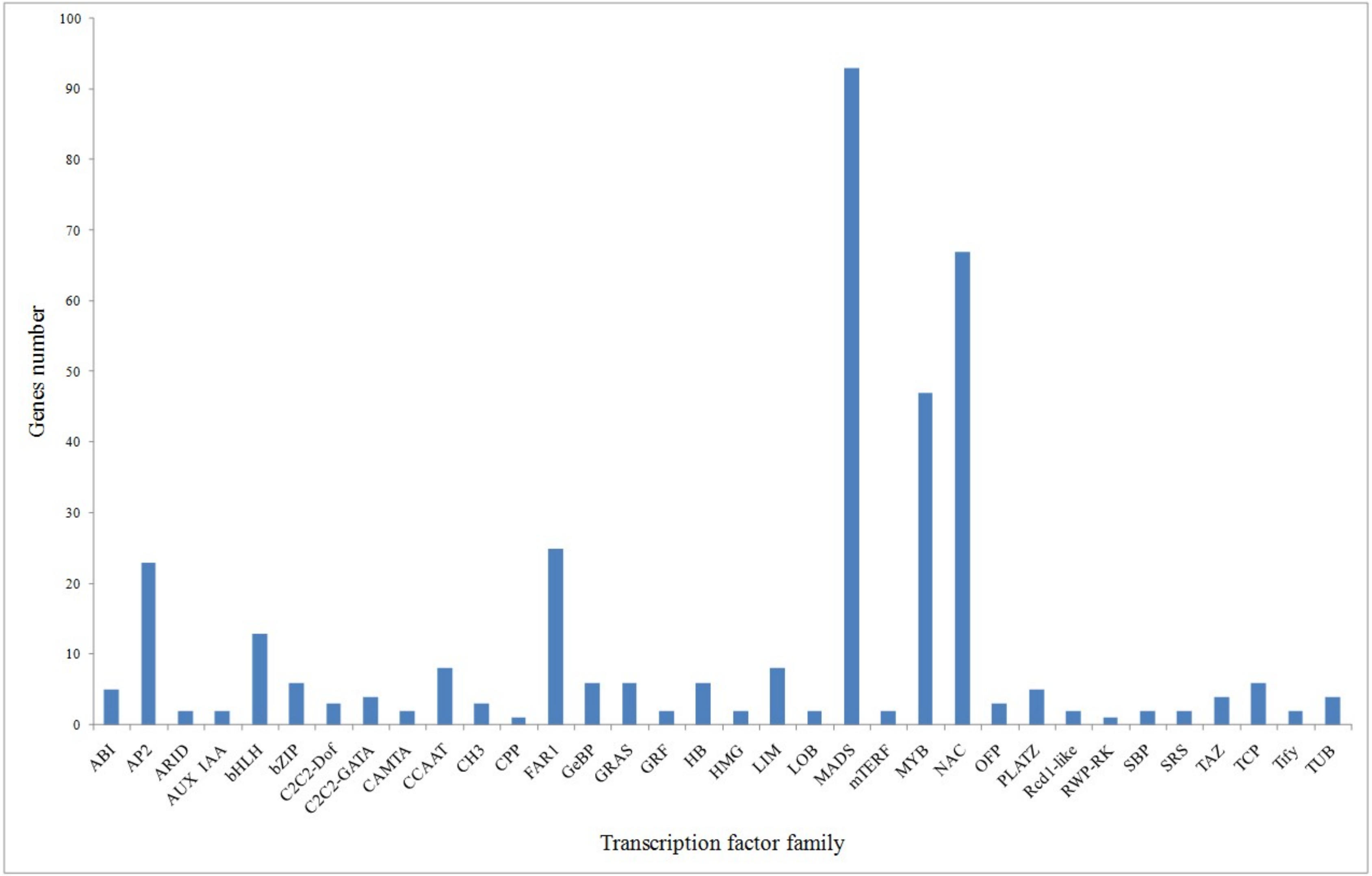

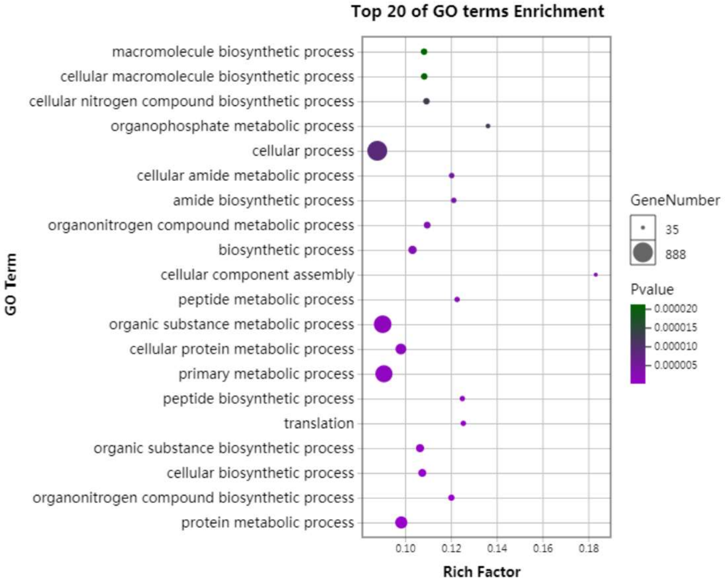


Biological process

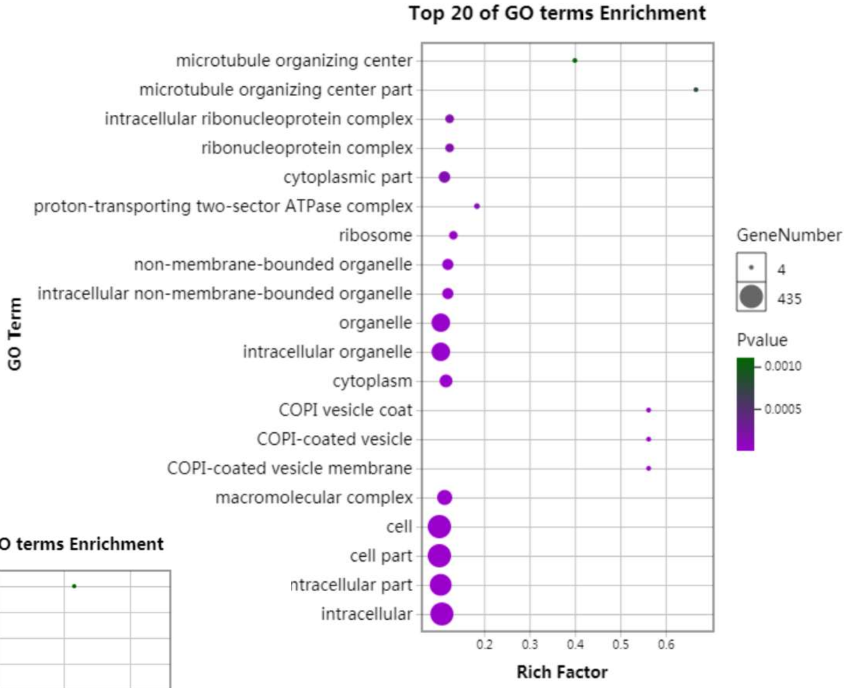


Cellular component

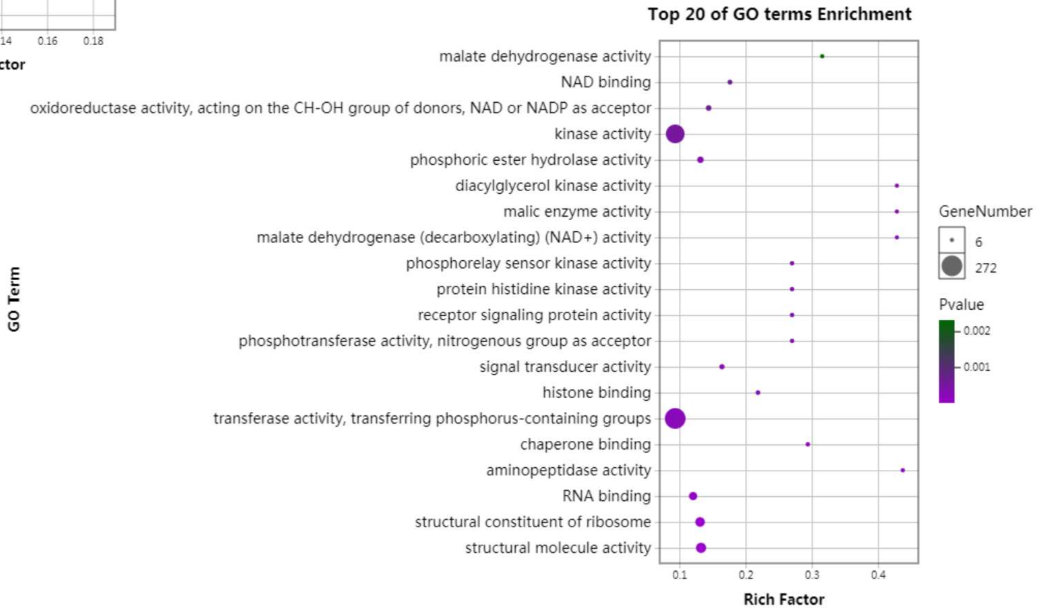


Molecular Function

**Supplementary Figure 7**

GO enrichments of duplicated genes after WGD in *H. cannabinus*

**Supplementary Figure 8** Relationship between leaf shape and fiber accumulation at the different stages of growth.

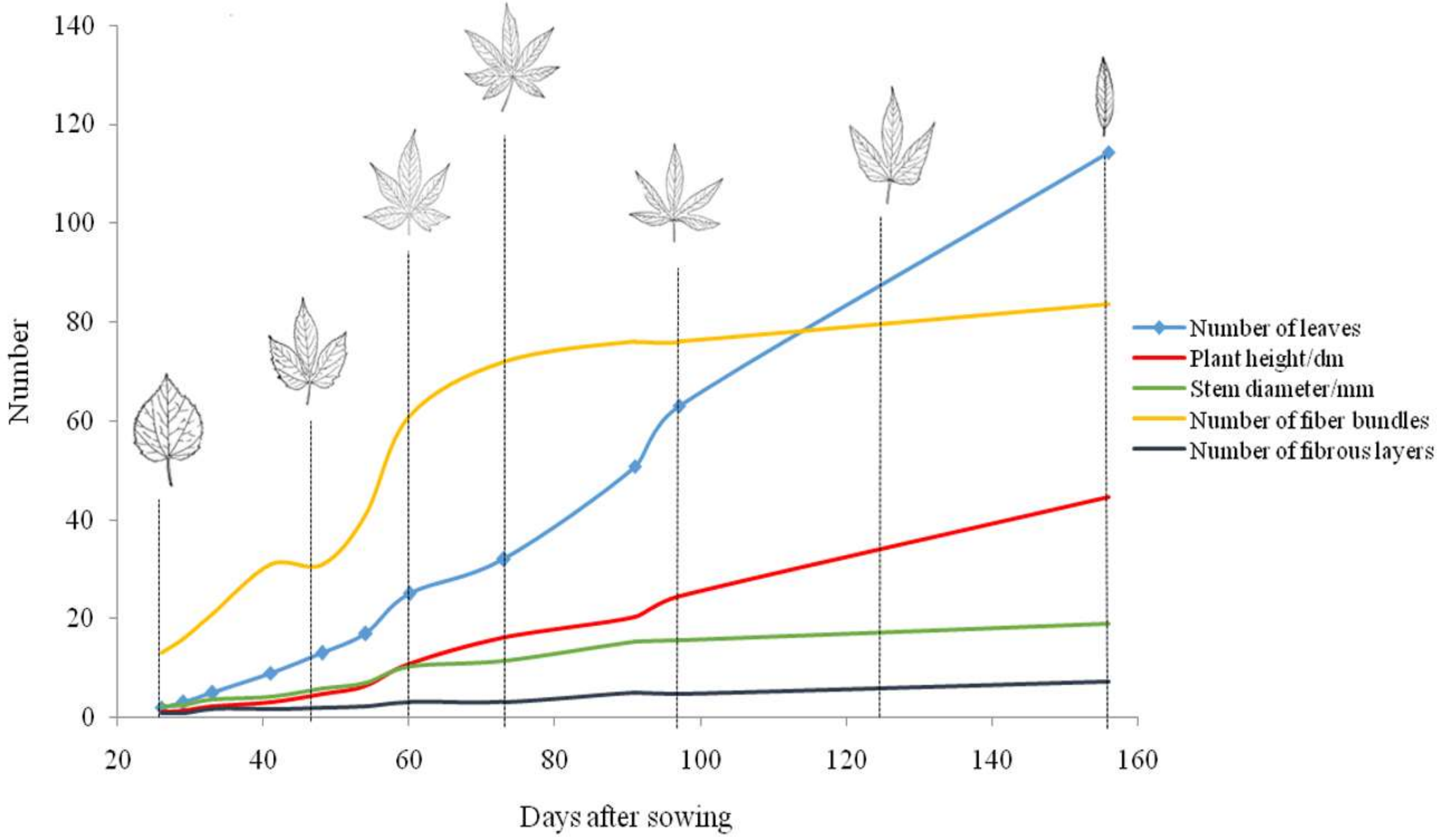

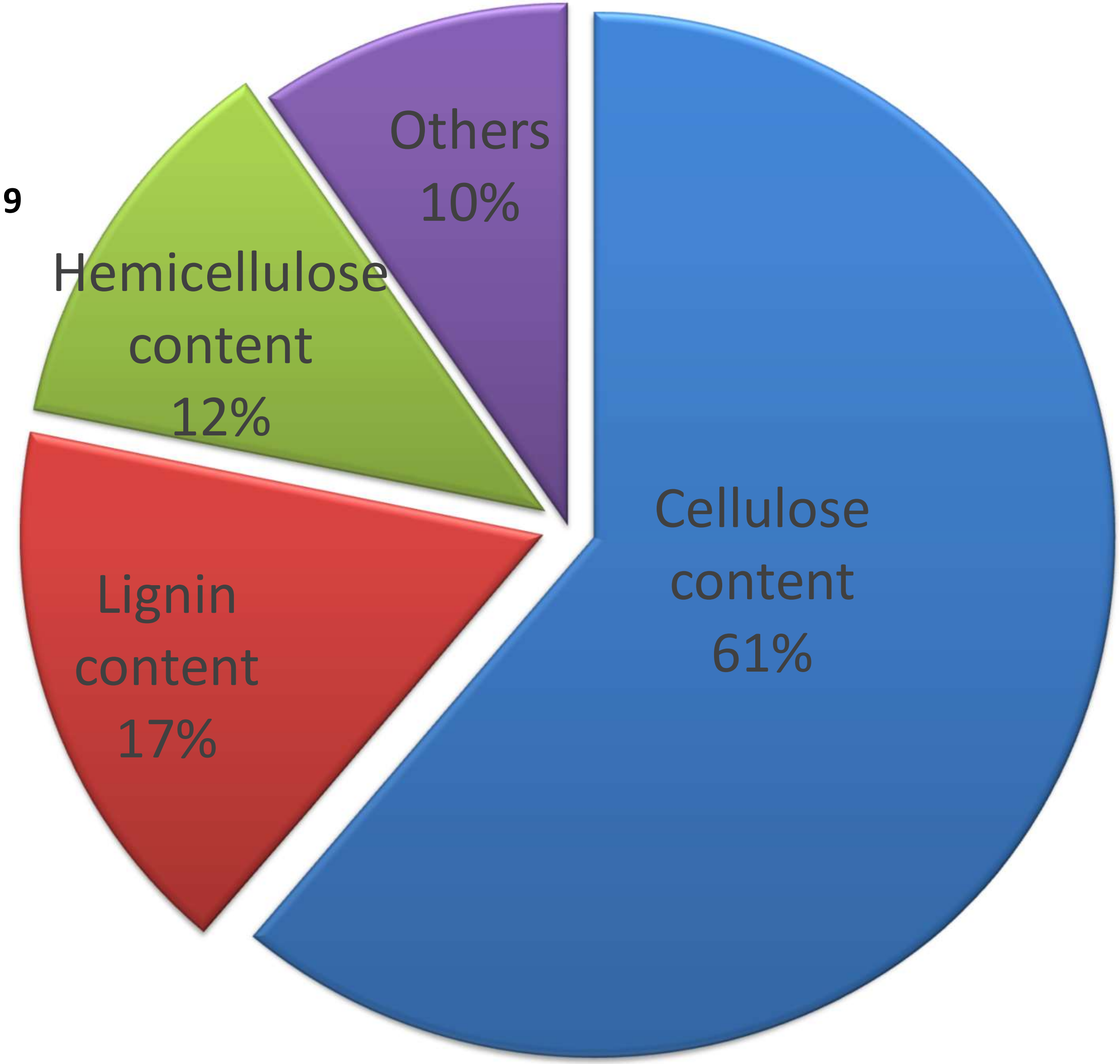


**Supplementary Figure 9**
Different chemical components of bast fiber in *H. cannabinus.*

**Supplementary Figure 10** BSA mapping of the candidate leaf shape gene (*HcLMI1*) in the population of ZZF2. BSA mapping showing the candidate leaf shape gene (*HcLMI1*) with a high ΔSNP-index value, a high SNP-index value in the bulked lobed-leaf sample, and a low SNP-index value in the bulked round-leaf sample in *H. cannabinus*

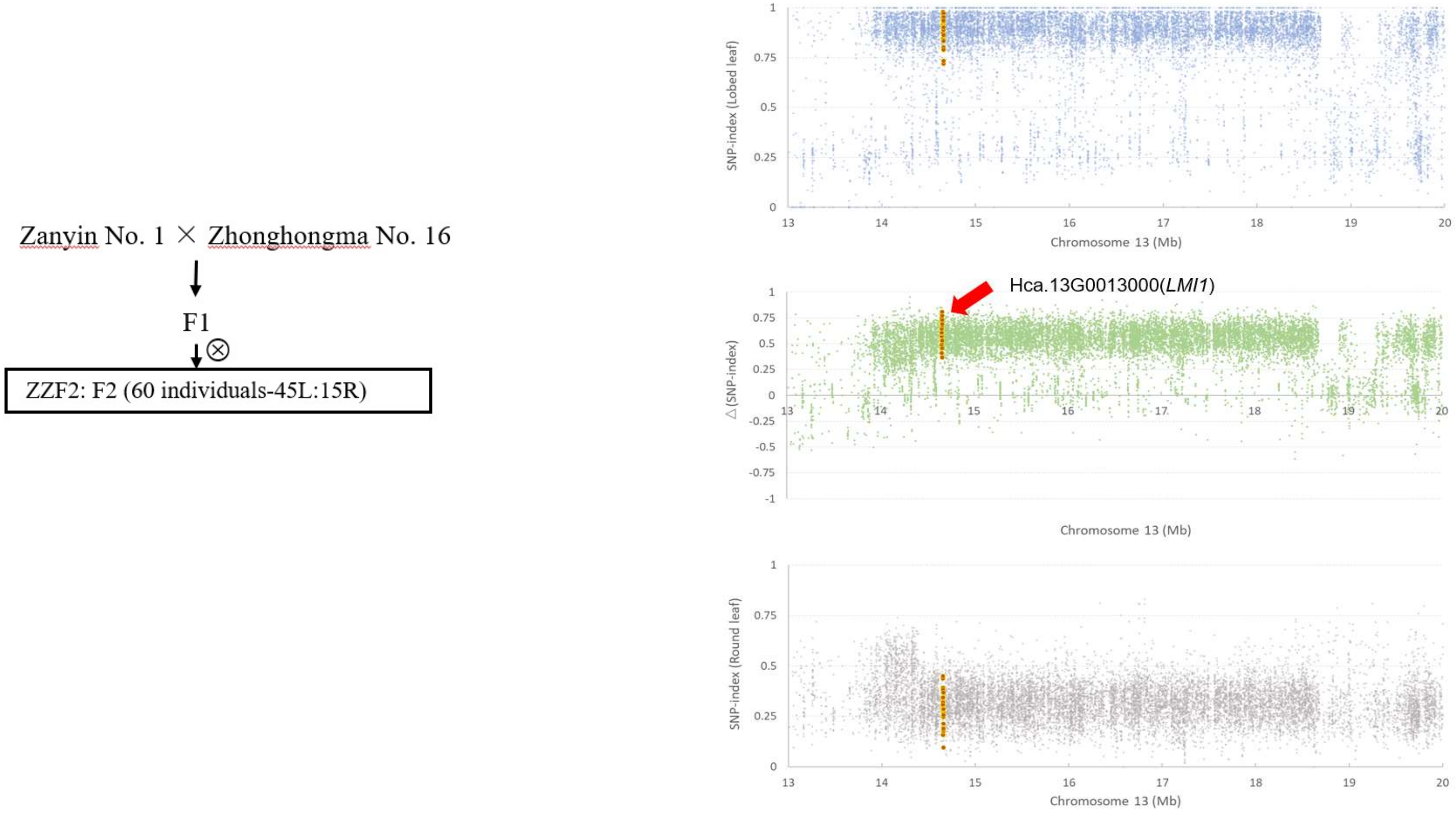

**Supplementary Figure 11** Development of near iso-genic lines using backcrosses

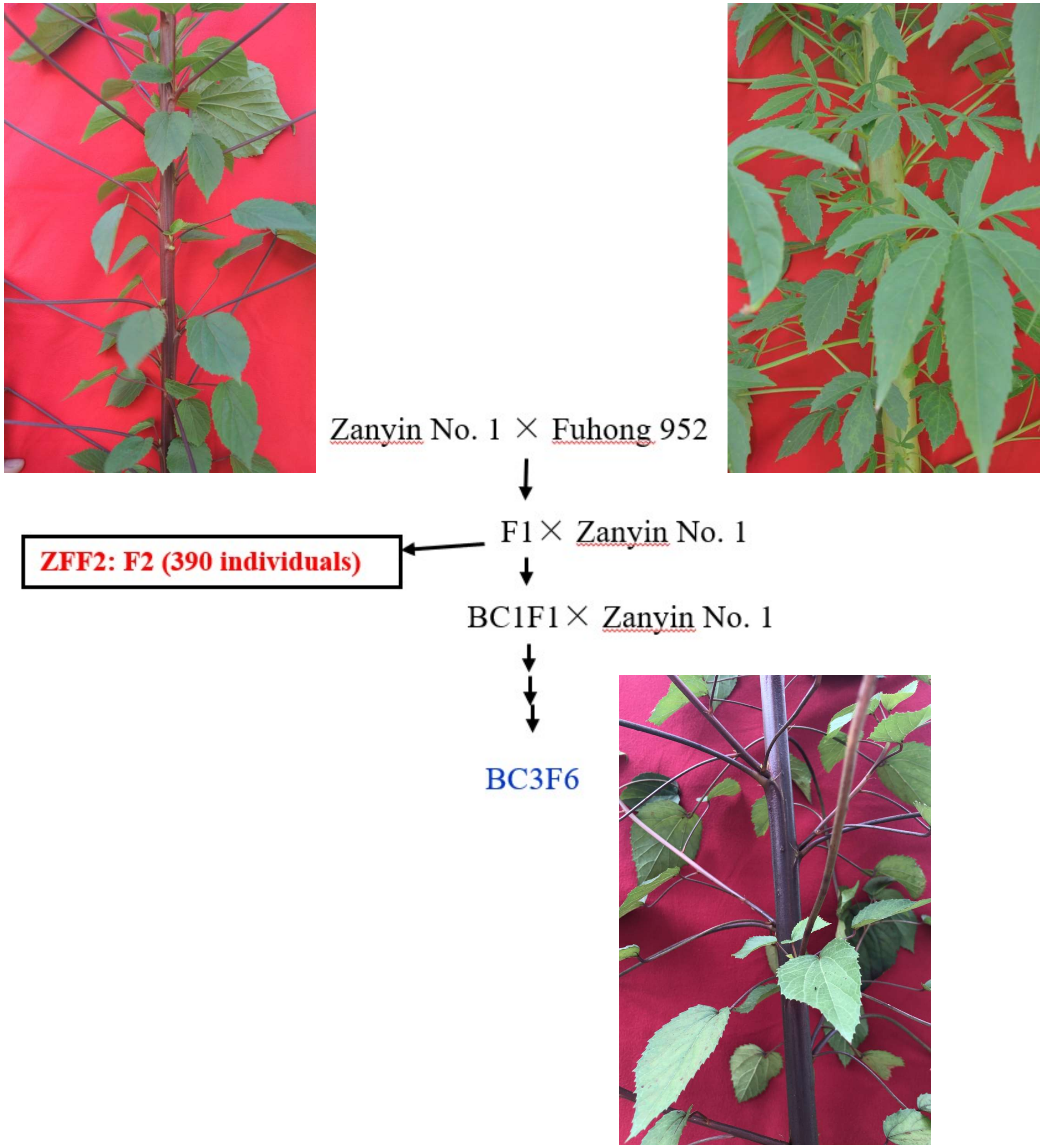

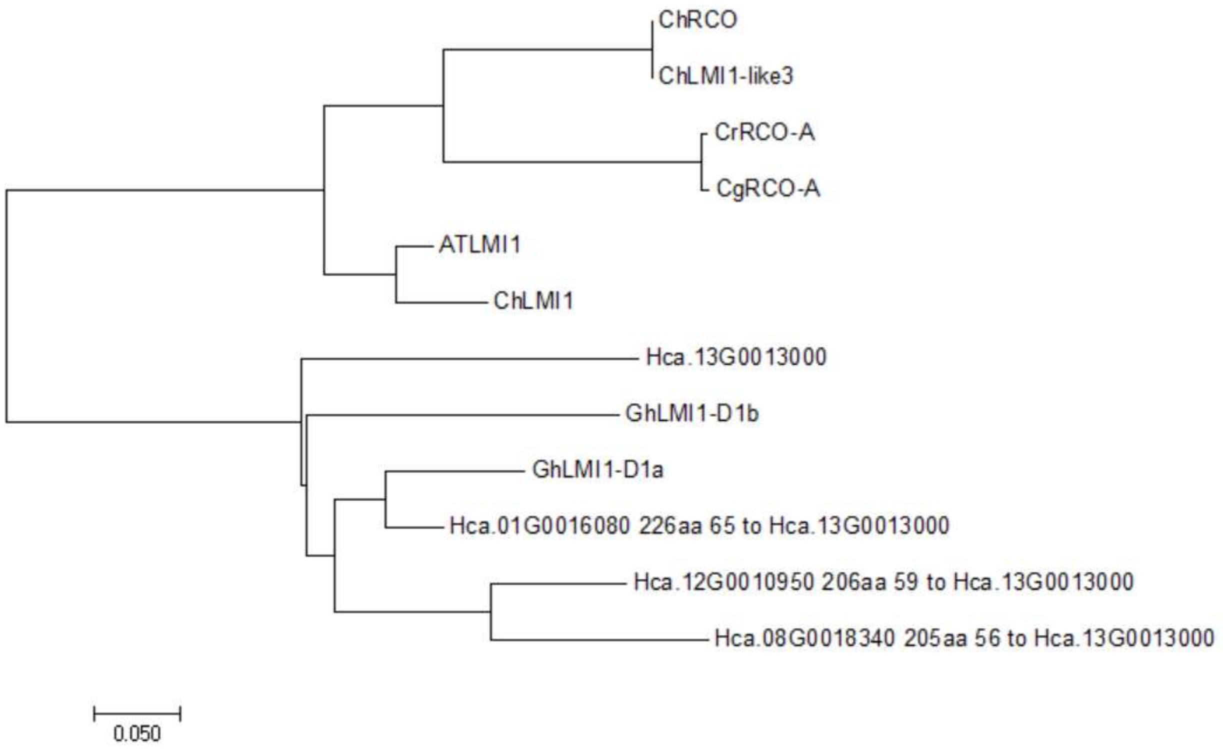


**Supplementary Figure 12** Evolutionary relationships of *LMI1*. *Ch: Cardamine hirsuta; Cr:Capsella rubella; Cg; Capsella grandiflora; Gh:Gossypium hirsutum.*

**Supplementary Figure 13** Expression analysis of genes from different tissues at the various stages in *H. cannabinus*

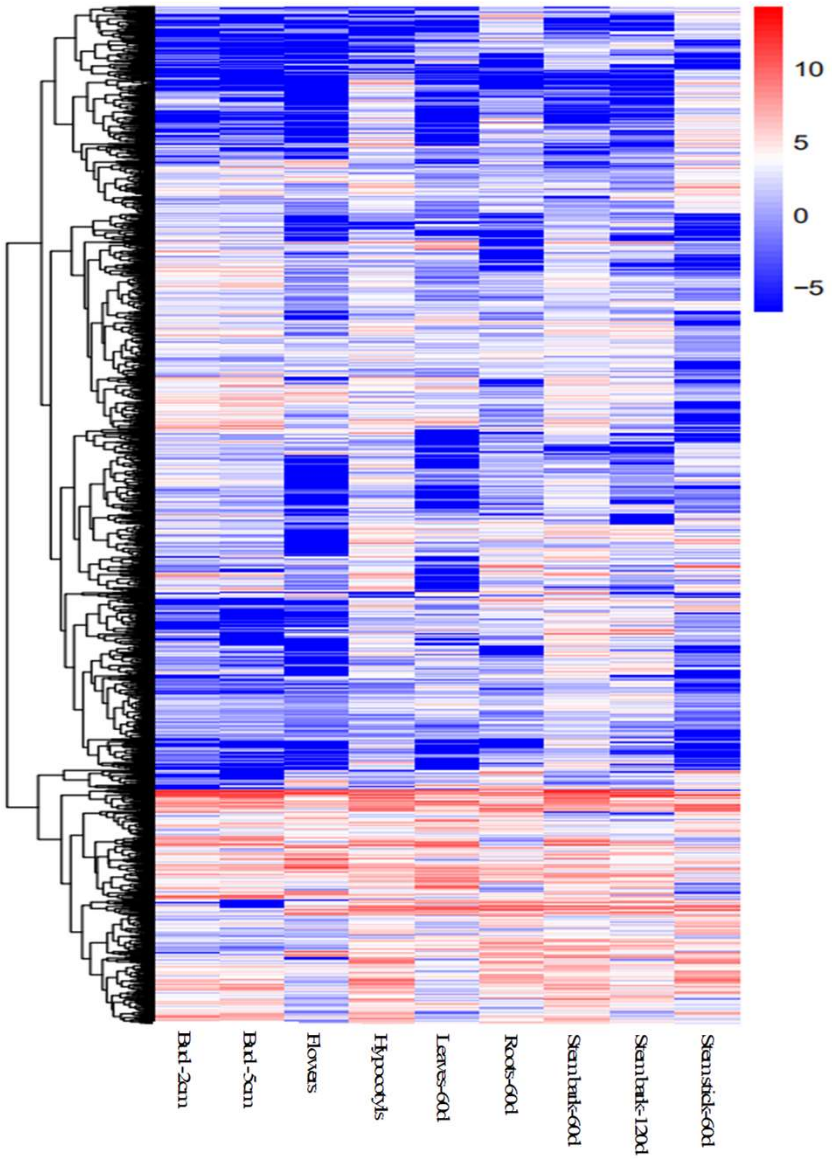

**Supplementary Figure 14** Comparison of copy numbers of genes involved in secondary cell wall enzymes between *Hca* and *Ath*. Comparison of relative RNA-Seq expression of secondary cell wall enzymes in leaves.60d and stem bark.60d. *Hca, H. cannabinus; Ath, A. thaliana*.

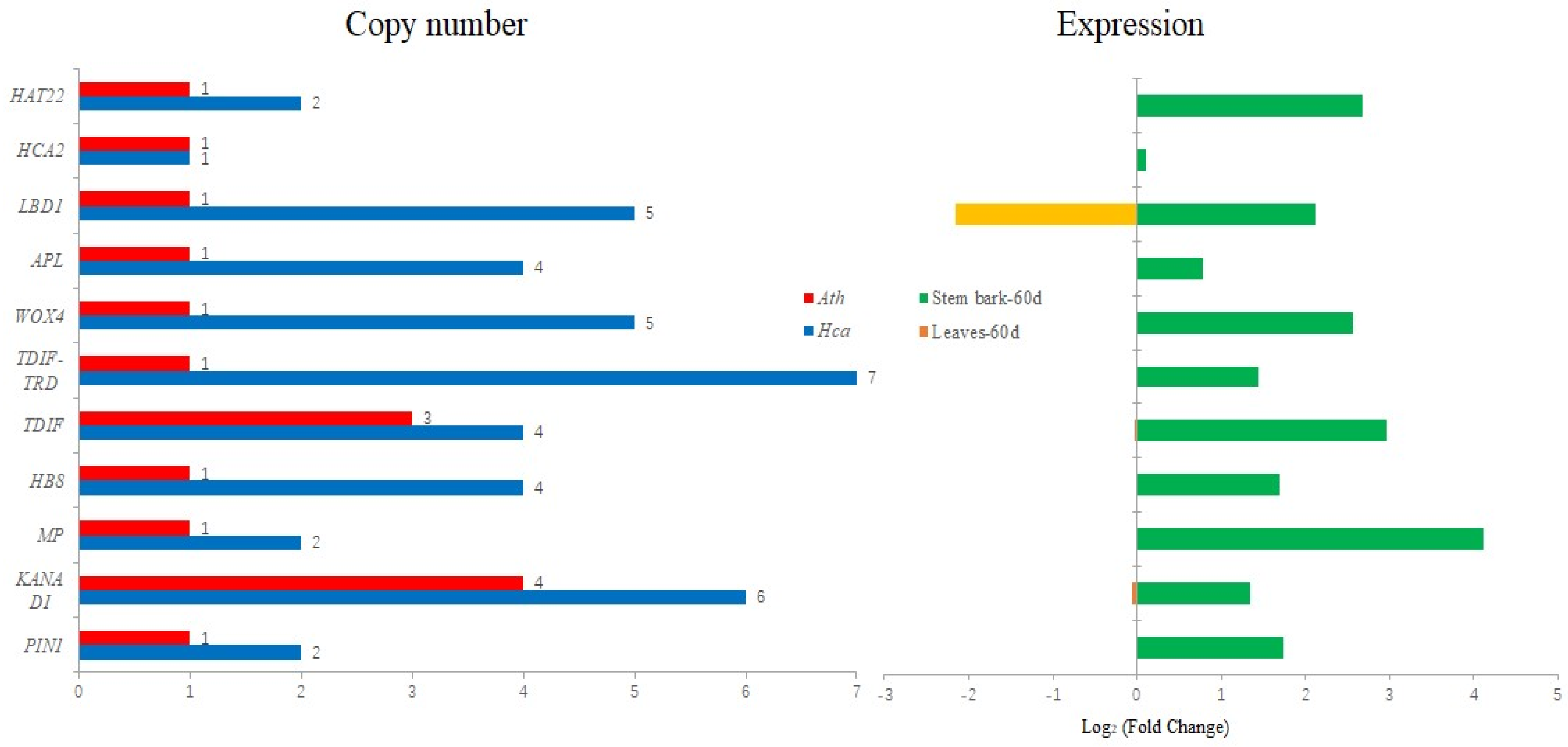

**Supplementary Figure 15** Synthenic analysis between *H. cannabinus* and the close related species *T. cacao*

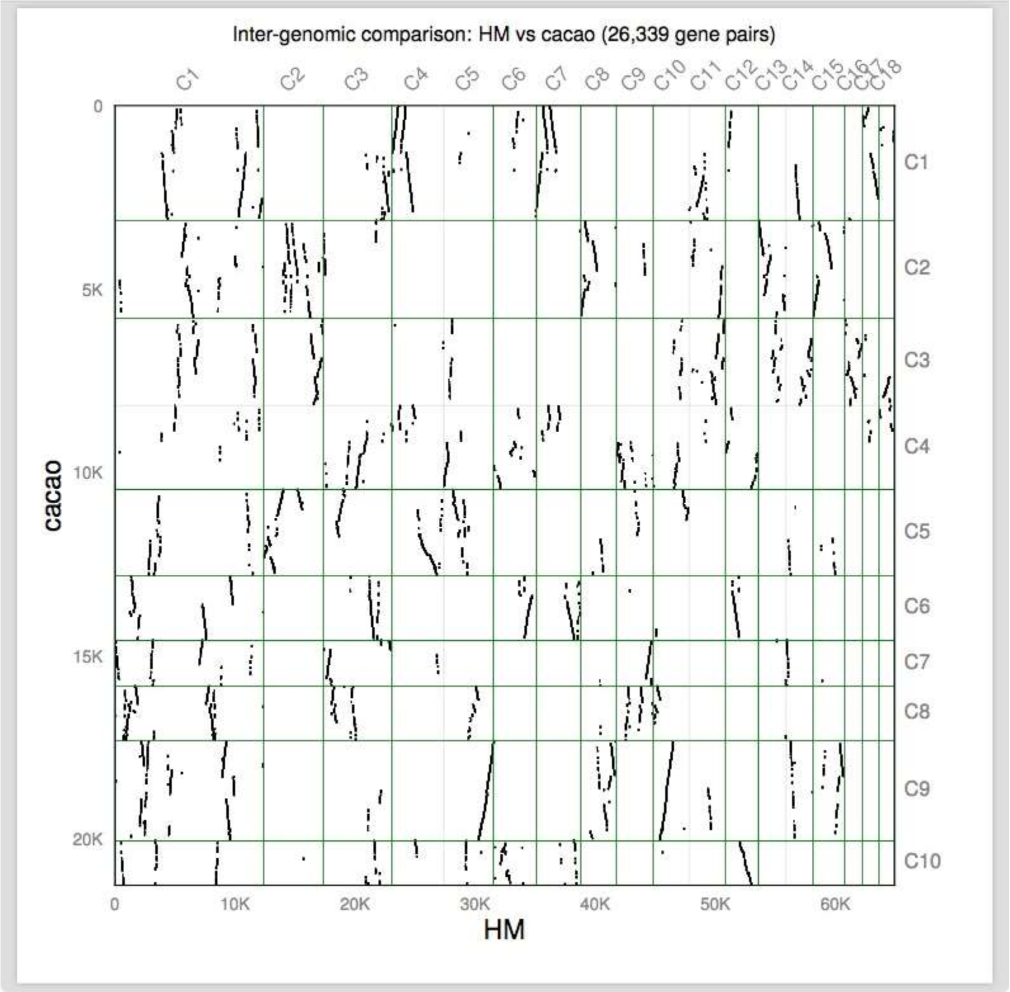

**Supplementary Figure 16** Phylogenetic analysis of cellulose synthase A (*CesA*) genes in *Arabidopsis thaliana* and *H. cannabinus.* Red circles represent up-regulation at stem-bark-60d, while blue one indicates up-regulation at leaves-60d.

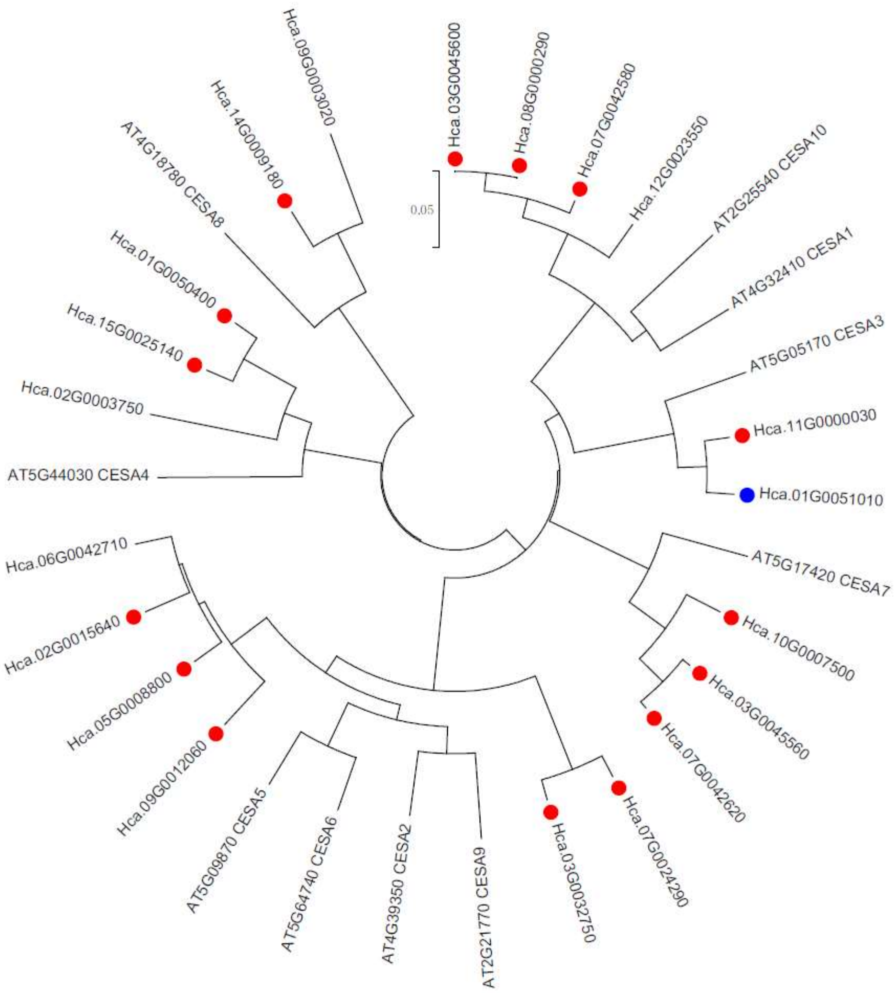

**Supplementary Figure 17** Genetic diversity and structure variations among 20 core kenaf cultivars based on SNPs.

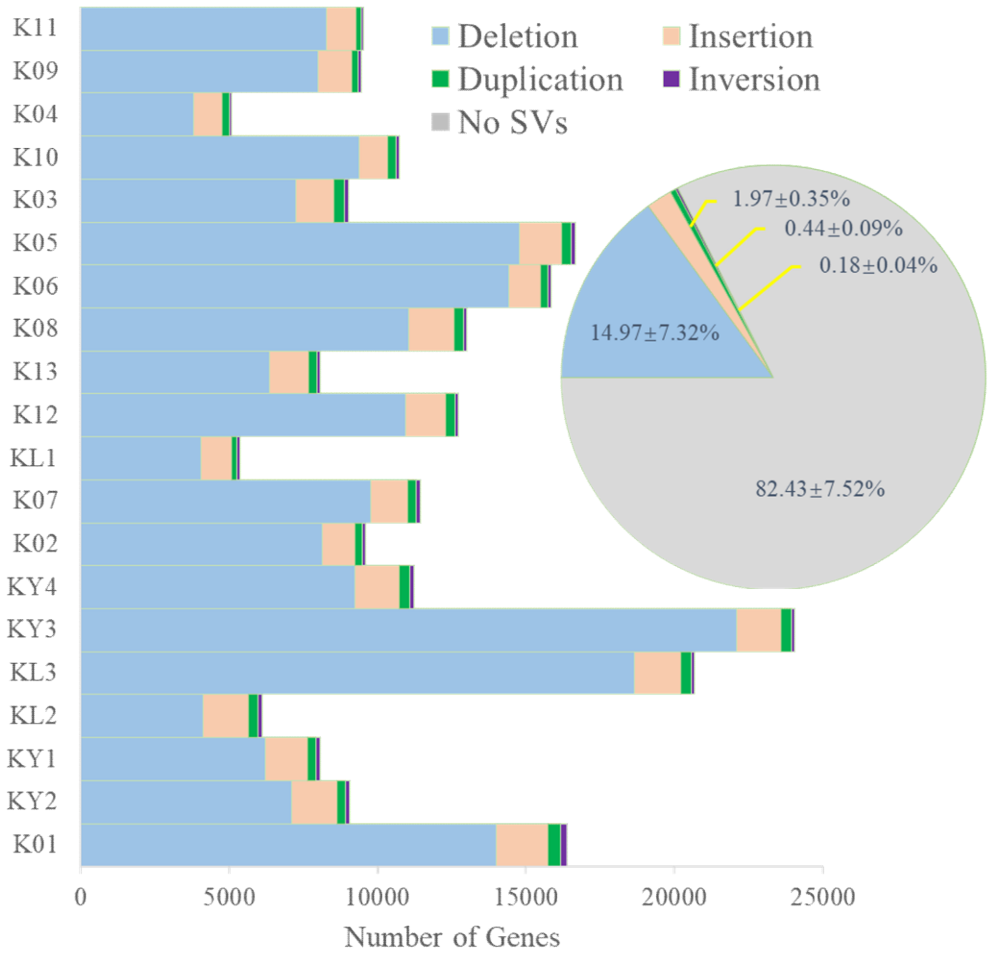

**Supplementary Figure 18** Number of genes with deletions of 20 core *H. cannabinus* cultivars

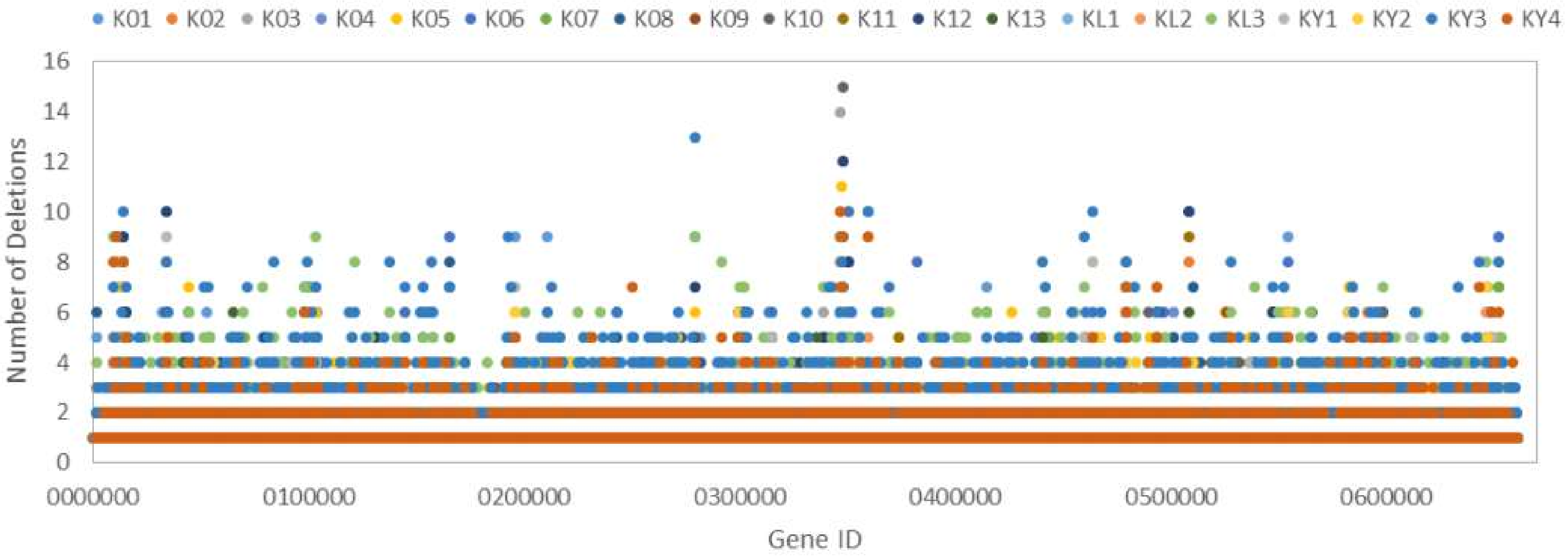

**Supplementary Figure 19** Number of genes with insertions of 20 core *H. cannabinus* cultivars

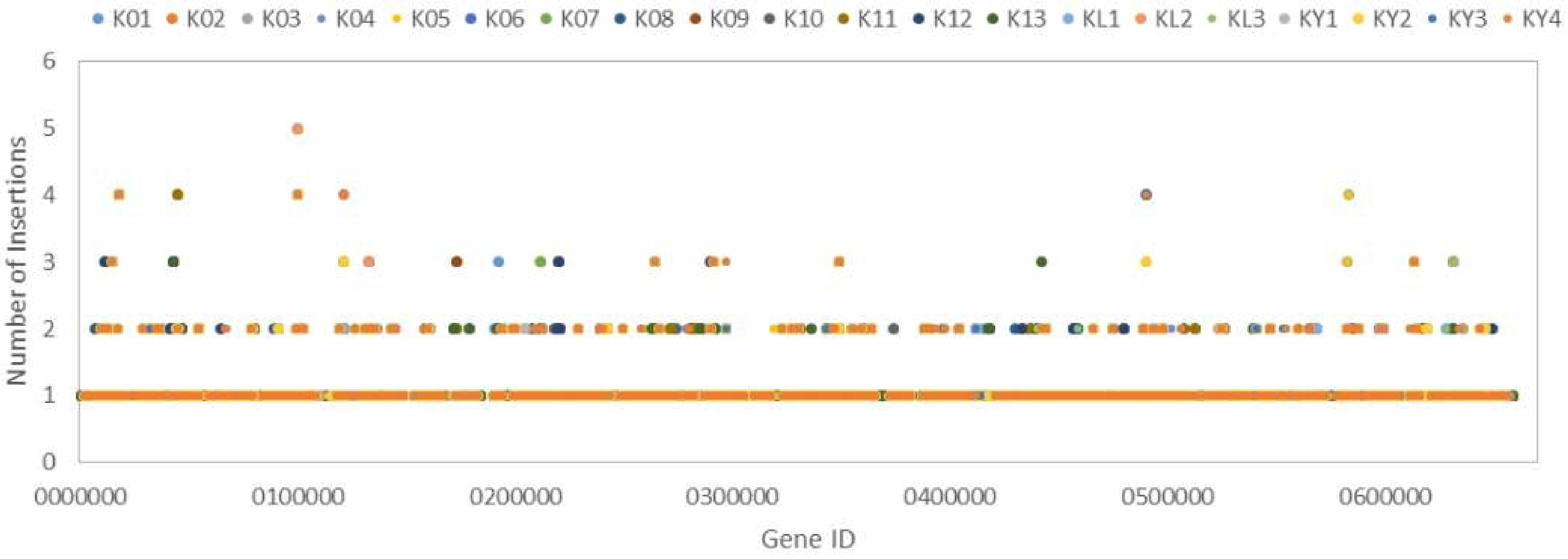

**Supplementary Figure 20** Number of genes with invertions of 20 core *H. cannabinus* cultivars

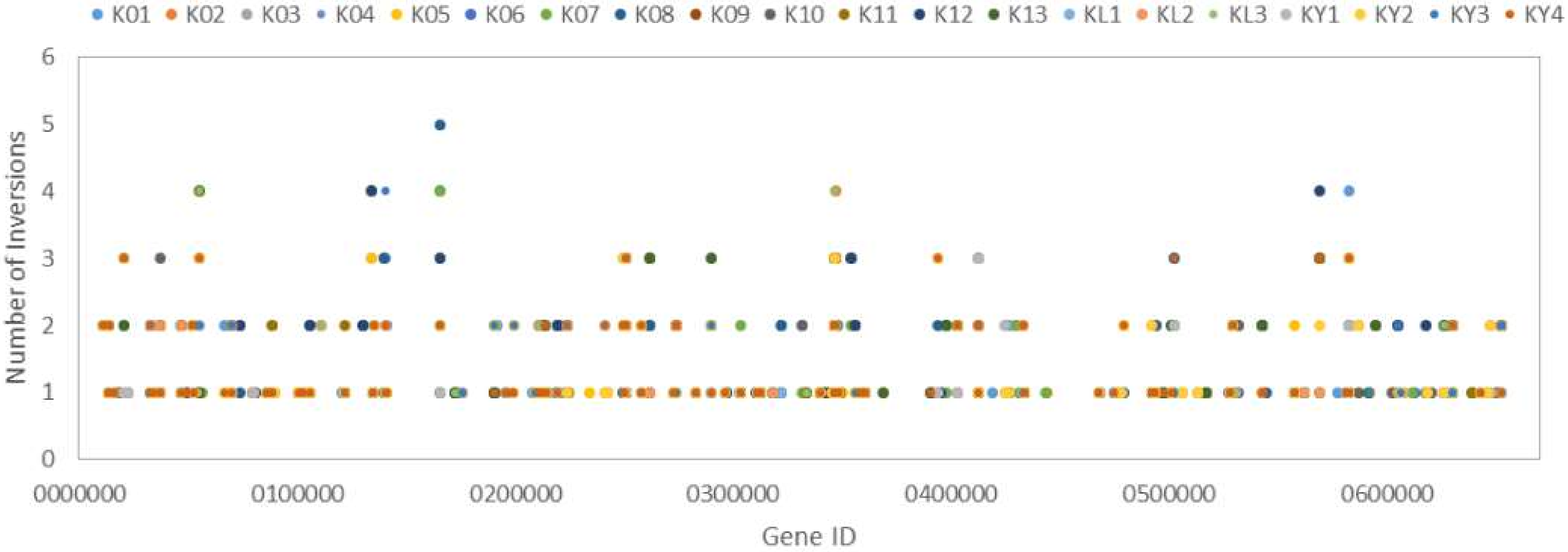

**Supplementary Figure 21** Number of genes with tandem duplications of 20 core *H. cannabinus* cultivars

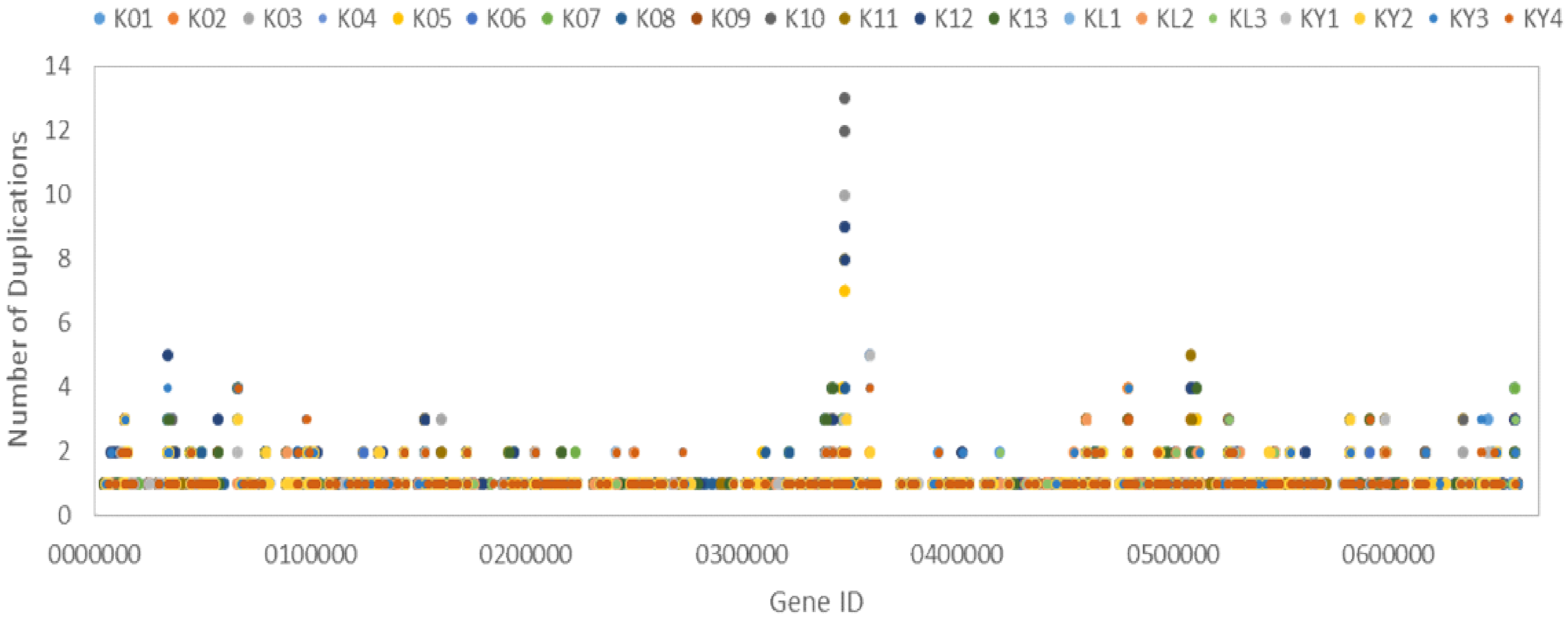

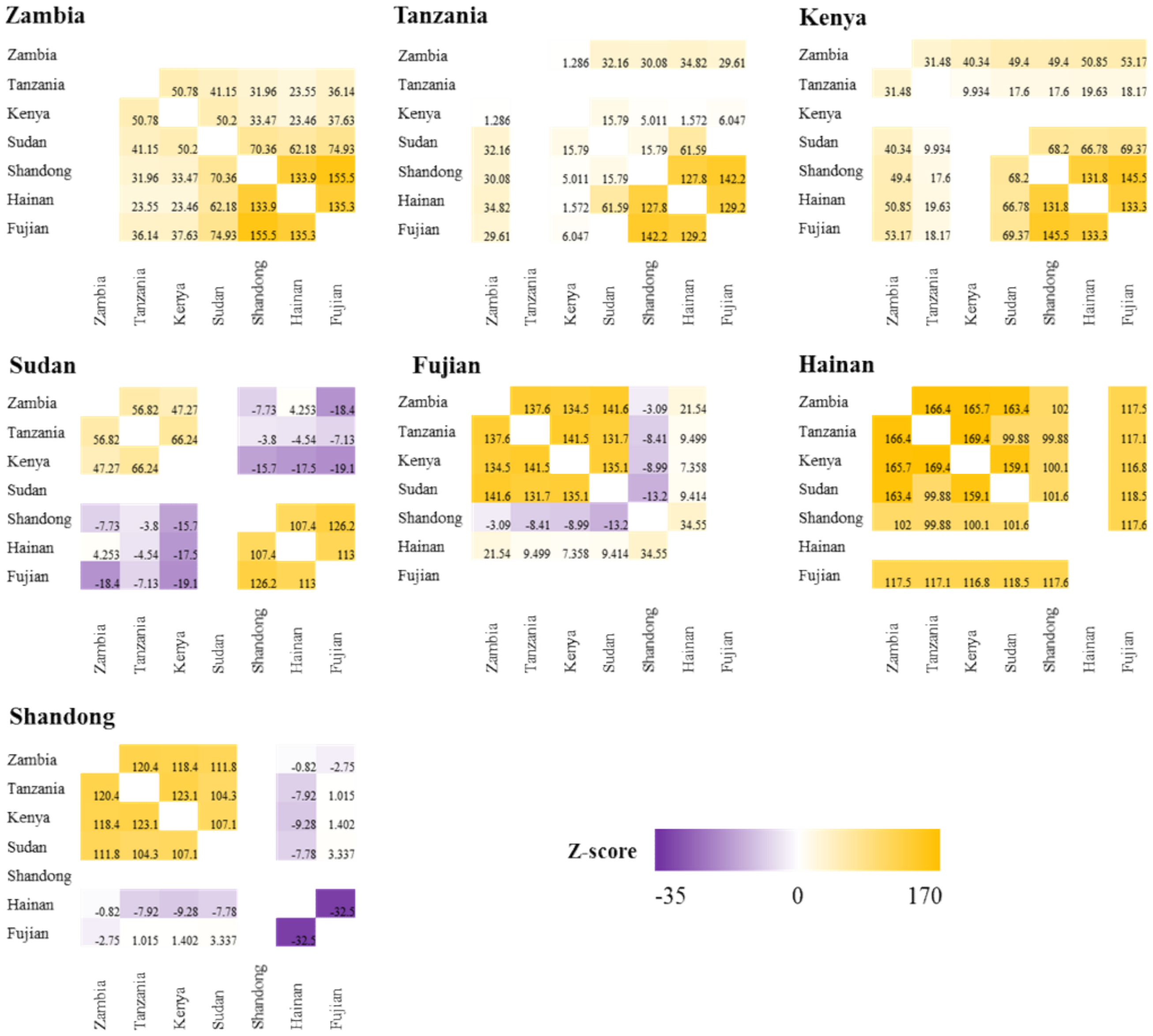

**Supplementary Figure 22** Heat maps for introgression detection among different geographical regions.
Introgression is significant if the Z-score is significantly negative.

**Supplementary Figure 23**
Selective sweeps scanning in 20 core *H. cannabinus* cultivars. Identification of candidate regions by the composite likelihood ratio (CLR) score across kenaf genome. Each chromosome was divided into 2,000 windows, and each dot represents one window. The red solid line indicates the candidate regions above the 1% cutoff outlier with significant deviations from neutrality, indicating recent selective sweeps.

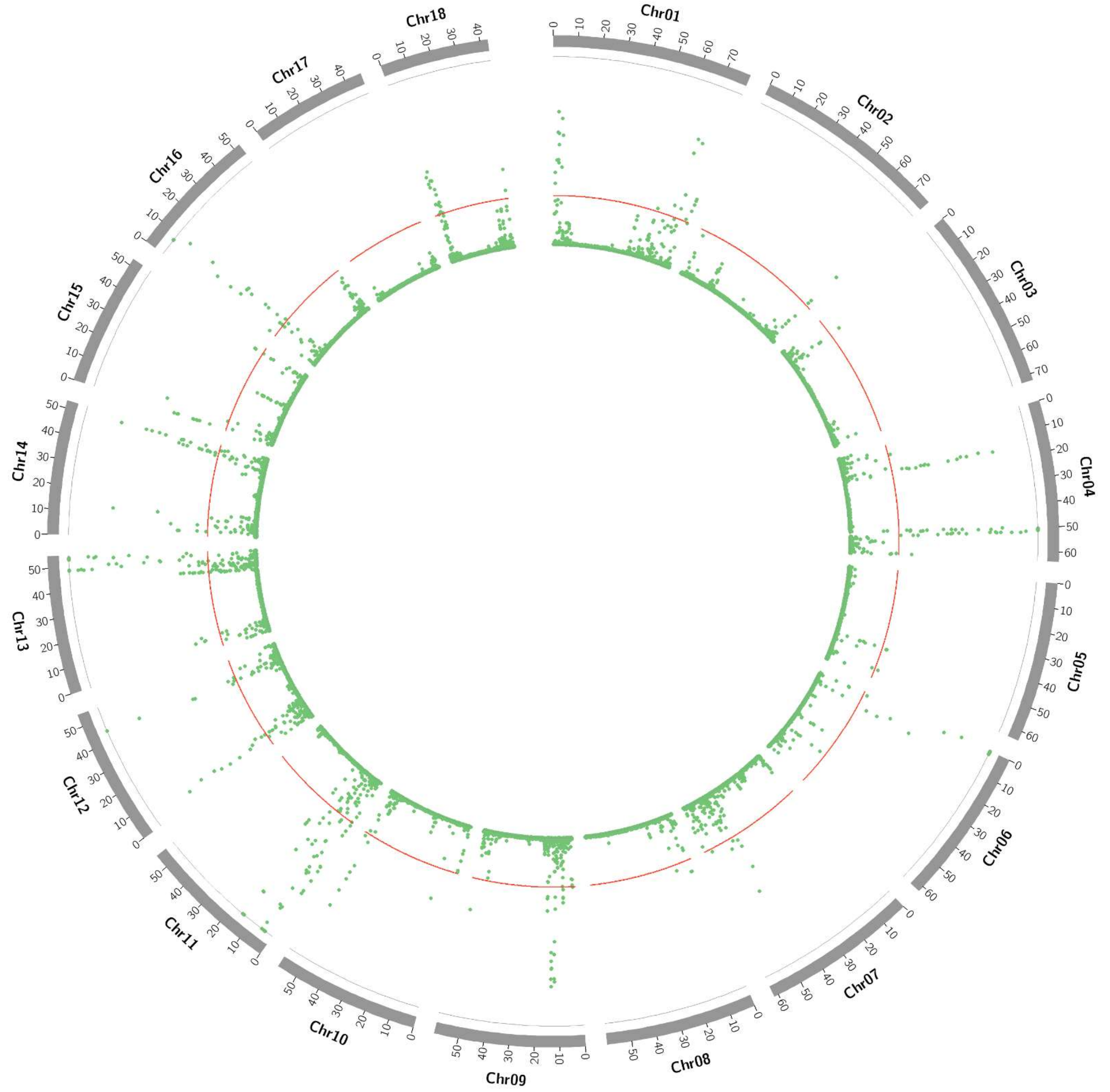

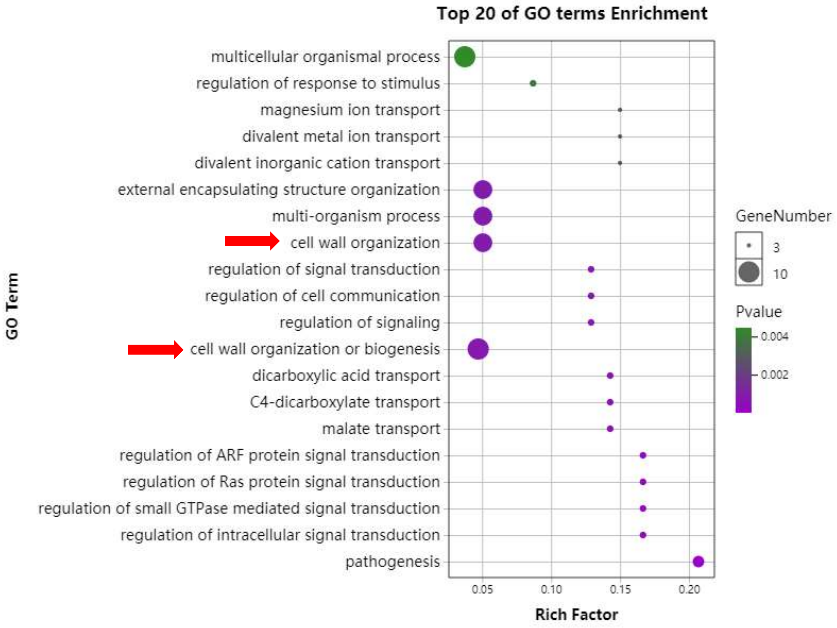


Biological process

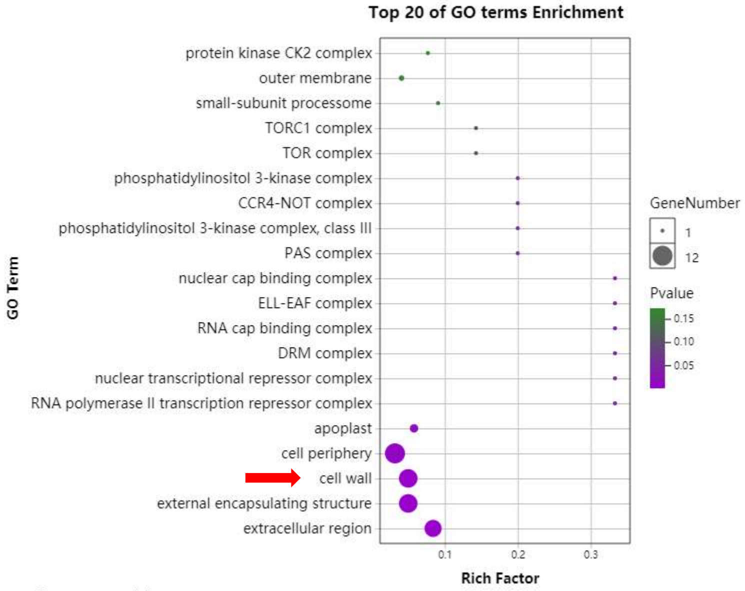


Cellular component

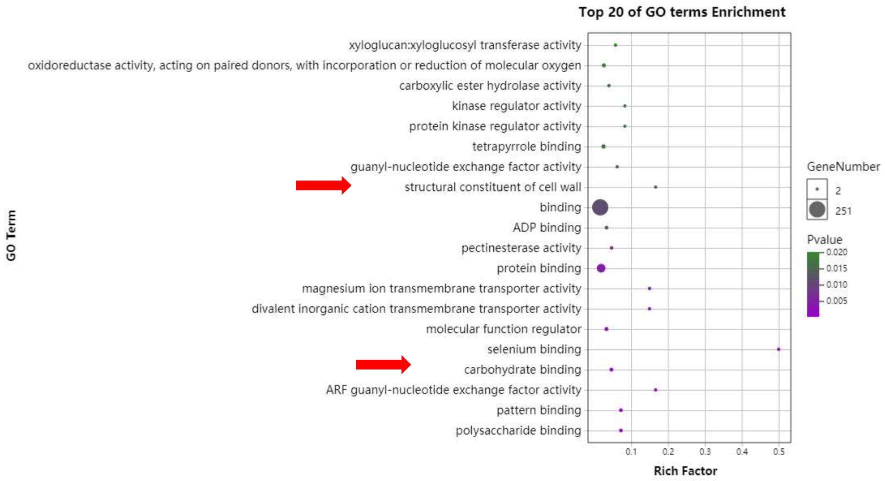


Molecular Function

**Supplementary Figure 24**

GO enrichments of selective swept genes in 20 core *H. cannabinus* cultivars
