# Supplementary material for: The genome of kenaf (Hibiscus cannabinus L.) provides insights into bast fibre and leaf shape biogenesis
Source: Plant Biotechnol J. 2020 Jan 30;18(8):1796–809. doi: 10.1111/pbi.13341 (PMC7336286; doi:10.1111/pbi.13341)
Supplement: Supplementary file 3 — Appendix S1 Materials and Methods. [file PBI-18-1796-s004.doc]

**Materials and Methods**

**1 Genome sequencing and assembly**

**1.1 Sample collection**

*Hibiscus cannabinus* cultivar Fuhong 952, which is a control cultivar in the official variety registry in China, is chosen for reference genome sequencing and assembly. The plants were grown at the farm of Fujian Agriculture and Forestry University, Fuzhou, China. And leaves were collected for genome sequencing from a single plant. To investigate the fiber development, we collected samples from different stages and tissues for RNA sequencing, including hypocotyls, roots, stem barks, stem sticks, leaves, buds and flowers.

**1.2 DNA extraction and library preparation**

DNA was extracted from leaf tissue of a single soil-grown plant using the Qiagen DNeasy Plant Mini Kit and subject to 500-bp paired-end (PE) libraries construction using the NEBNext Ultra DNA Library Prep Kit for Illumina sequencing. Sequencing was performed using Illumina HiSeq X10 platform (Illumina, San Diego, CA, USA), which were conducted at Center for Genomics and Biotechnology (Fuzhou, China) and Beijing Biomarker Technology Co., Ltd (Beijing, China). 150 bp PE reads were trimmed by Trimmomatic v0.36 with default parameters. For the Pacbio sequencing, DNA was extracted from leaf tissue of the same single plant at Genomics and Biotechnology (Fuzhou, China) and Nextgenomics Technology Co., Ltd (Wuhan, China). More than 50 µg of sheared and concentrated DNA was applied to size-selection by BluePippin system. ~20 kb SMRTbell^TM^ libraries were prepared according to the released protocol from PACBIO Company. A total of 15 Single-Molecule Real-Time (SMRT) cells were run on Pacbio Sequel system with P6-C4 chemistry and appropriate 77 Gb raw data were generated.

**1.3 RNA extraction and library preparation**

RNA was extracted using the Illumina TruSeq™ RNA Sample Preparation Kit (Illumina, San Diego, CA, USA) following the manufacturer’s recommendations. For the construction of RNA-seq libraries, 2 µg of total RNA was processed using the TruSeq RNA Sample Preparation kit (Illumina) followed by sequencing on the Illumina HiSeq ×10 platform at Center for Genomics and Biotechnology (Fuzhou, China) and Beijing Novogene Biological Information Technology Co., Ltd. (Beijing, China).

**1.4 Genome assembly and assessment**

The contig level assembly was performed on full PacBio long reads using CANU package, which is a successor of Celear Assembler with an increased performance on continuity and decreased running time. CANU version 1.5 was used for self-correction and assembly with parameter corOutCoverage=100, ovbMemory=8g, maxMemory=500g, maxThreads=48, ovsMemory=8-500g, ovsThreads=4, oveMemory=32g on a SGE grid. Subsequently, the draft assembly was polished using Arrow (https://github.com/PacificBiosciences/GenomicConsensus). To increase the accuracy of assembly, illumina short reads were recruited for further polishing with the Pilon program.

The accuracy and completeness of the assembly were assessed by various approaches. We first de novo assembled the RNA-seq reads using Trinity and estimated the expression with RSEM . Transcripts with FPKM < 1 and iso-percentage < 3% were removed and the remaining transcripts were blasted against the genome assembly. We found that 441,970 out of 485,096 (91.11%) transcripts were able to align to at least one contig with 90% sequence identity. When the transcripts were filtered by length, we observed that 97.38%, 99.21% and 99.80% of transcripts, with a minimum length of 500 bp, 1000bp and 2000bp respectively, were aligned to assembly. We further used CEGMA version 2.5 and BUSCO version 3 pipelines . Our results showed that 234 (94.35%) gene models out of the 248 ultra-conserved core eukaryotic genes (CEGs) in CEGMA analysis and 1,375 (95.5%) out of 1440 conserved genes in BUSCO analysis were completely recalled in our assembly. We also mapped the illumina short reads to the Pacbio-only assembly. 121.75 million (99.3%) out of 122.6 million reads were mapped in the assembly and covered 96.97% of assembled size. These results indicate a high-quality assembly with an extremely low proportion of misassemblies and high level of completeness.

**1.5 Hi-C library preparation and sequencing**

Hi-C libraries were created from tender leaves of *H. cannabinus* as described Xie et al. Briefly, the leaves were fixed with formaldehyde, lysed, and then the cross-linked DNA digested with *HindIII* overnight. Sticky ends were biotinylated and proximity-ligated to form chimeric junctions, that were enriched and then physically sheared to a size of 500-700 bp. Chimeric fragments representing the original cross-linked long-distance physical interactions were then processed into paired-end sequencing libraries and 212 million of 150bp paired-end Illumina reads were produced. The paired-end reads were uniquely mapped onto the draft assembly contigs which were grouped into 18 chromosome clusters, and scaffolded using Lachesis software with tuned parameters.

**1.6 High density genetic map construction**

To conduct a high density genetic map, a segregating population had been generated from a cross between Fuhong 952 × Zanyin No. 1, in which Fuhong 952 is the most widely cultivated cultivar in China for whole genome sequence and Zanyin No. 1 is a pure line introduced from Zambia. Progeny of 131 F_2_ individuals were used for map construction. Twenty elite *H. cannabinus* cultivars and this segregating population were planted at the experimental farm of Fujian Agricultural and Forestry University, Fuzhou, China on May 1st, 2016. Genomic DNA from the parental accessions and 131 F_2_ individuals was extracted from 30-day-old seedlings using a modified cetyltrimethyl ammonium bromide method.

Paired-end sequencing reads of the 131 progenies of F_2_ population passing Illumina’s quality control filter were aligned, using the Burrows–Wheeler aligner (v0.6.1) to map to the H. cannabinus genome v1(HM.pilon.tmp.fasta). The raw unfiltered SNPs and indels was generated using the Genome Analysis Toolkit (GATK, version3.5), Realigner Target Creator and Indel Realigner protocol for global realignment of reads around InDels from the sorted BAM files.

Variants calling used the GATK Unified Genotyper set concurrently for all 131 progenies samples with default filtering settings. Low coverage and repetitive variants were removed from the raw VCF file containing 6,814,191 unfiltered SNPs and InDels.

Raw VCF files were filtered with the GATK standard filter method. The variants with<5 or >30×coverage, mapping qualities (PHRED scores) <25 and number of alleles >2, missing rate >0.1 were removed from the raw vcf file for further analysis. After filtering, 218,328 SNPs and indels were retained as the input for constructing the genetic map.

The custom scripts were used to generate a set of 3,828 markers for genetic linkage map construction. The linkage map was constructed by makers with less than 20% missing data of each sample implemented in MapMaker/exp3.0. We filtered the markers with segregation ratios significantly deviated from 1:3 (A:H) ratio in the F_2_ population. The parameters LOD values from 3 to 12 (with 1 increment) and recombination frequency from 0.25 to 0.05 (with 0.05 decrement) were used for map construction. The centimorgan distances were calculated by Kosambi mapping function . To order the markers, we used the recombination counting and ordering (RECORD) algorithms. The regression algorithm was used for calculating to generate the linkage groups.

**2 Transcriptome sequencing and gene annotation**

**2.1 Assembly of RNA-seq data**

Selected RNA-seq samples were imported into Trinity de novo assembly and genome-guided assembly pipelines with default parameters . RSEM was used to calculate transcript abundance . Transcripts with FPKM < 1 and iso-percentage < 3 % were removed from further analysis. The filtered transcripts were subject to PASA program for construction of comprehensive transcripts. PASA is able to take advantage of the high sensitivity of reference-based assembly while leveraging the ability of de novo assembly to detect novel transcripts.

**2.2 Annotation of protein-coding genes**

The PASA-assembled transcripts described above were used for training. The nearly “full-length” transcripts were evaluated by comparing with UniProt plant protein database and proteins that were covered at least 95 % were retained as candidates. Then *ab initio* gene predictors, including SNAP , GENEMARK and AUGUSTUS , were each trained with those selected proteins.

We used MAKER pipeline to generate the comprehensive set of protein-coding genes. MAKER pipeline integrate multiple tiers of coding evidence, including *ab initio* gene prediction, transcript evidence and protein evidence. A total of 80,612 gene models were identified in the first round of MAKER run without masking the customized repeat library. We then performed an extensive filtering of TE-related genes and removing of short proteins (less than 30 aa). Finally, a set of 66,004 protein-coding genes were kept for downstream analysis.

**2.3 Identification of repetitive elements**

We first customized a *de novo* repeat library of the genome using RepeatModeler (http://www.repeatmasker.org/RepeatModeler/), which can automatically execute two *de novo* repeat finding programs, including RECON (version 1.08) and RepeatScout (version 1.0.5) . The consensus TE sequences generated above were subject to RepeatMasker (version 4.05) to identify and cluster repetitive elements. Unknown TEs were further classified using TEclass (version 2.1.3) .

To identify tandem repeats within the genome, the Tandem Repeat Finder (TRF) package (version 4.07) was used with the modified parameters of “1 1 2 80 5 200 2000 –d -h” in order to find high order repeats. Telomeres and centromeres were identified based on the .dat output files above. Repeat sequences with more than 10 monomers ‘AAACCT’ were identified as telomeres. For centromeres identification, we used a similar method described in *Oropetium thomaeum* genome . The largest repeat arrays were identified and clustered as centromeres.

To further investigate LTRs, we applied LTR_retriever pipeline , which is able to integrate results from public programs, e.g. LTR_FINDER and LTRharvest, and efficiently remove false positives from the initial predictions. The predict LTRs were further classified into intact and non-intact LTRs and the insertion time was estimated as T=K/2μ (K is the divergence rate and μ is the neutral mutation rate) using the scripts implemented in LTR_retriever package.

**2.4 Identification of small RNAs**

We used miRDeep-P pipeline to identify known microRNAs. Briefly, the public microRNAs collected from miRBase 21 (<http://www.mirbase.org/>) were mapped to the reference genome using bowtie with a maximum of three mismatch. Reads that mapped to genome more than 15 times were removed. Candidates were extracted from the mapping file and then subject to RNAfold (version 2.43) to predict secondary structure. MiRDeep-P employed the signature distribution of small RNAs and also developed a plant-specific scoring system to identify potential microRNAs. Totally, we identified 132 microRNAs in *Hibiscus cannabinus* genome.

**3 Differentially expressed genes (DEGs) analysis**

The FPKM (reads per kb per million reads) value was calculated for each gene. Differential gene expression analysis using edgeR.FDR (False Discover Rate)<0.05 and log_2_(fold change)>0 were identified significantly up-regulated gene. FDR <0.05 and log_2_(fold change)<0 were set as the threshold for significant down-regulated gene.

**4 Genome comparison and evolution**

**4.1 Identification and comparison of gene families**

For gene family analysis, BLASTP was used to compare all annotated *Hibiscus cannabinus* protein sequences against a protein data set of 7 sequenced plant sepecies(*Arabidopsis thaliana*, *Theobroma cacao*, *Gossypium* *hirsutum*, *Gossypium* *raimondii*, *Linum usitatissimum*, *Populus trichocarpa*, *Oryza sativa*) with an E-value cutoff of ≤ 10^-5^. The proteins were clustered using OrthoMCL to difine gene families which included both paralogs and orthologs. The number of gene families in each species and genus was calculated based on the composition of OrthoMCL clusters.

**4.2 Phylogenetic analysis**

The single copy genes of each species were identified use PYTHON script base the OrthoMCL clustersand performed multiple alignments of protein sequences with MUSCLE, the result of alignments was selected to construct a phylogenetic tree. Divergence times between *Hibiscus cannabinus* and other species were estimated using MEGA.

**4.3 Genome speciation event deduced from orthologous pairs**

The orthologous pairs was slected base the OrthoMCL clusters, which were used to calculate the Ks (the number of nonsynonymous substitutions per nonsynonymous site) by YN00 in the PAML^11^ package.

**4.4 Identification of whole genome duplication**

The predicted protein sequences from *H. cannabinus* were blast against itself with a maximum of 5 hits. MCscanX was used to analyze genome synteny with default parameters. The pairs of duplicated genes generated by WGD were identified if the two genes shared high level of collinearity with at least 5 nearby genes being in a same synteny block.

**5 Real-time PCR**

The RNA extracted from leaf, root, stem bast and stem stick was used for RT-qPCR. cDNA was obtained using a reverse transcription kit (TaKaRa PrimeScript™). To confirm the expression level using FPKM (Fragments Per Kilobase of exon per Million fragments mapped), genes involved in leaf shape were chosen as cases for PCR validation. RT-qPCR was performed using an ABI 7500 ﬂuorescence quantitative PCR instrument. Each reaction contained cDNA (1 μL), gene-specific primers (0.5 μL, 10 μmol L−1) and 2× SYBR Green Master Mix (10 μL) in a final volume of 20 μL. The *H. cannabinus* action gene was served as the endogenous control. Real-time quantitative PCR analysis was as follows: 95 °C for 10 min, followed by 40 cycles of 95 °C for 15 s, 55°C for 20s and 72°C for 30s. All experiments were used in triplicate for each sample and relative gene expression levels were calculated using the 2^−ΔΔCT^ method.

**6** **Kenaf population genomics**

**6.1*Variants calling***

In total, 20 core kenaf accessions were resequenced for population genomics analyses. The raw pair-end reads were trimmed to remove the adaptors and low-quality bases using Trimmomatic after quality control by FastQC. The reads were filtered with a sliding window of size 7, with average Phred score scale = 20 within the window. The trimmed reads were mapped to the reference genome using Bowtie2 with default parameters. The mapped reads were sorted, and duplicated reads were removed using SAMtools. The Realigner Target Creator and Indel Realigner programs from the Genome Analysis Toolkit (GATK) package were used for global realignment of reads around indels from the sorted BAM files. The HaplotypeCaller of the GATK was used to estimate the SNPs and Indels for putative diploids using the default parameters. The distribution of calling depths (DP) of each raw variant were estimated as a criterion for variants filtering. Low depths and repetitive variants were removed from the raw VCF file if they had DP < 2 or DP > 40, minQ < 20. We allowed the variants sites with maximum-missing rate as 20%. These filtering strategies reduced the raw unfiltered variants to the working set of 2,697,218 variants (SNPs and Indels). SnpEff v3.6c was used to assign variants effects based on gene models from reference genome annotation.

**6.2 Genome-wide genetic diversity and LD decay estimation**

The high confidence filtered 2,697,218 variant set was used for population genomics statistics estimations. Population statistics of SNP density, π and Tajima’s D were calculated from the filtered VCF file in 200-kb sliding window for π, SNPs density, Tajima’s D and *F*-statistics (Weir & Cockerham *Fst*) in VCFtools. One-sample t-test was used to evaluate the statistically significant if Tajima’s D deviating from zero. Genome-wide Linkage disequilirium (LD) was calculated and LD decay curve was fitted in PopLDdecay (https://github.com/BGI-shenzhen/PopLDdecay) with default parameters.

**6.3 PCA, phylogeny and population structure**

Principal component analysis (PCA) was performed using the GCTA software on the filtered 2,697,218 variants. The input Plink binary files are transformed from the filtered VCFs file using VCFtools and PLINK. The top two principal components were used for assigning the 20 accessions. Bi-allelic and polymorphic 2,143,286 SNPs were used for reconstructing the phylogenetic relationships among 20 accessions using SNPhylo software. Before tree construction, we filtered and pruned the SNPs (with MAF< 0.05, and missing rate > 0.3, LD threshold = 0.1). The multiple consensus sequences were aligned using MUSCLE. ML trees were constructed using maximum likelihood method by running DNAML programs in the PHYLIP package. In addition, BS tree was constructed by bootstrapping (bootstrap =10000) analysis using PHANGORN package. Figtree v.1.4 (http://tree.bio.ed.ac.uk/software/figtree/) was used for to visualize the trees. The optimal ancestral population stratification was estimated from the same variants set with ADMIXTURE using ancestral population clusters K = 1~20 and choosing the population with smallest cross-validation error. DISTRUCT was used to plot the population stratification results for K = 1 through K = 20.

**6.4 Demography history of effective population size (*N_e_*)**

Site frequency spectrum (SFS) of 20 cultivated accessions were estimated using ANGSD. We calculated the site allele frequency likelihood based on the SAMTools genotype likelihood model at all sites, and then obtained a maximum likelihood estimate of the SFS using the Expectation Maximization (EM) algorithm. The SFS was then used for estimating the population demography history using software Stairway plots with 200 bootstrap iterations. Because of the variation of molecular substitution rate for wild kenaf and relative’s populations, we used a range of 1.5e-9, 6.5e-9, and 10e-9 as the mutation rate parameters for analysis. As the generation time of wild kenaf is one year, we carried out the estimation using one-year generation time when doing Stairway plots.

**6.5 Genomic-wide selective sweeps scanning**

In terms of genomic-wide selection, we detected the absolute selective sweeps in kenaf population with grid size of 20 k using the SweeD software. The CLR (composite likelihood ratio) statistic was used as the criteria of recent selective sweeps by significant deviating from the neutral site frequency spectrum (SFS). The SFSs were calculated using ANGSD. The identification of candidate sweep regions by the CLR scores are scanned across the kenaf reference genome with each chromosome dividing into 2,000 windows in SweeD. The candidate sweep regions were identified as those exceeding the top 1% of CLR scores that significant deviations from neutrality. Genes overlapping sweep regions (including ±2kb flanking regions of each boundaries) were treated as genes putatively under selection. The swept genes were then performed the functional GO annotation and enrichment analysis in Blast2Go v4.1. We used swept genes as tested gene sets, the whole gene models of kenaf as references. The significance of enrichments was valued using the Fisher’s exact test.

**7 VIGS**

Gateway technology (Fernandez-Moreno et al., 2013; Hartley et al., 2000) is used to construct the vector in this experiment. Its operation process is as follows: The ORF of LMI was cloned from kenaf leaves, and the target fragments (~250bp) needed for the experiment was recovered. The recovered fragments were transferred to pDONR207 vector by BP reaction, and then transformed into E. coli to obtain positive clones. After sequencing, the required positive bacteria were screened and the recombinant plasmids were extracted. The target fragment was transferred from the recombinant vector to the expression vector TRV2 by LR reaction. Agrobacterium strains containing target fragments were obtained by Agrobacterium transformation screening and PCR validation. When combined with the TRV1 vector of the bipartite TRV VIGS system, this treatment was named TRV: LMI1.

In addition to the TRV: LMI experimental treatment, one negative controls were used as before (Tuttle et al., 2008). TRV: TRV1 consisted of an empty TRV2 vector and TRV1, which could controls for effects of the inoculation process. Because silencing from TRV can fluctuate over time(Senthil-Kumar and Mysore, 2011) and TRV VIGS is very sensitive to temperature and humidity(Fu et al., 2006), a TRV: CLA1 treatment (TRV: CLA1, consisting of TRV1 plus TRV2 containing a silencing fragment for CLA1, is capable of spreading throughout the plant.) that influence chloroplast development was used as a visible marker to ensure that environmental conditions were suitable for VIGS and to time the phenotyping of LMI knockdowns.

**References**
